# Identification of hit compounds with anti-schistosomal activity on *in vitro* generated juvenile worms in cell-free medium

**DOI:** 10.1371/journal.pntd.0009432

**Published:** 2021-05-25

**Authors:** Nermina Vejzagić, Ulrich Fabien Prodjinotho, Nagwa El-Khafif, Ruili Huang, Anton Simeonov, Thomas Spangenberg, Clarissa Prazeres da Costa

**Affiliations:** 1 Institute for Microbiology, Immunology and Hygiene, Technische Universität München, Munich, Germany; 2 Center for Global Health, TUM School of Medicine, Technische Universität München, Munich, Germany; 3 Theodor Bilharz Research Institute, Mahad Al Abhas Al Bahari, Warraq Al Arab, El Warraq, Giza Governorate, Egypt; 4 Division of Pre-clinical Innovation, National Center for Advancing Translational Sciences, National Institutes of Health, Rockville, Maryland, United States of America; 5 Global Health Institute of Merck, Ares Trading S.A. (a subsidiary of Merck KGaA Darmstadt Germany), Eysins, Switzerland; Texas Biomedical Research institute, UNITED STATES

## Abstract

**Background:**

Anthelminthic treatment options against schistosomiasis are limited. The current treatment relies almost exclusively on a single drug, praziquantel (PZQ). As a consequence, the development of resistance to PZQ and limited activity of PZQ against earlier development stages are respectively a risk and a limitation to achieving the goals of the new WHO roadmap towards elimination. For the discovery of new chemical starting points, the *in vitro* drug screening on *Schistosoma mansoni* (*S*. *mansoni*) against newly transformed schistosomula (NTS) is still the most predominant approach. The use of only NTS in the initial screening limits sensitivity to potential new compounds which are predominantly active in later developmental stages. Using our recently described highly standardized, straightforward and reliable culture method that generates high rates of juvenile worms, we aimed to repurpose a subset of the National Center for Advancing Translational Sciences (NCATS) Pharmaceutical Collection (340 compounds) to identify new hits with an *in vitro* worm culture assay.

**Methodology/Principal findings:**

Cercariae were mechanically transformed into skin-stage (SkS) schistosomula and continuously cultured for 3–6 weeks to the liver stage (LiS). A commercial source of serum was identified, and decrease of NTS/well along with optimal drug testing conditions was established to test compounds on early and late LiS worms. The library was screened in 96-well format assays using praziquantel (PZQ) as a positive control. Primary screening allowed a 5.9% hit rate and generated two confirmed hits on adult worms; a prophylactic antianginal agent and an antihistaminic drug.

**Conclusion:**

With this standardized and reliable *in vitro* assay, important *S*. *mansoni* developmental stages up to LiS worms can be generated and cultured over an extended period. When exposed to a subset of the NCATS Pharmaceutical Collection, 3 compounds yielded a defined anti-schistosomal phenotype on juvenile worms. Translation of activity on perfused adult *S*. *mansoni* worms was achieved only for perhexiline (a prophylactic antianginal agent) and astemizole (an antihistaminic drug).

## Author summary

Schistosomiasis continues to be a major public health problem, mainly in developing countries. Although there have been some advances in finding new drugs, praziquantel is still the drug of choice. Certainly, one of the most important advances in the search for new treatments was the ability to *in vitro* transform cercariae and to grow schistosomula in culture. To reduce animal use in future drug discovery efforts (3Rs), we optimized a previously established reliable and robust *in vitro* cell-free culture system for the generation of liver-stage worms that we applied to the screening of a compound library stemming from the National Center for Advancing Translational Sciences (NCATS) Pharmaceutical Collection.

## Introduction

Schistosomiasis or bilharzia is caused by infection with trematodes of the genus *Schistosoma* and leads to chronic and debilitating disease [1]. Schistosomiasis is one of the most important neglected tropical diseases (NTD) with over 200,000 deaths each year due to the consequences of the disease [2]. According to WHO, in 2018, it was estimated that at least 290.8 million people required preventive treatment for schistosomiasis, with more than 97.2 million people receiving treatment [2]. Therefore, it is important to globally eliminate schistosomiasis as a public health problem over the next decade [3,4]. Implementation of several strategies, such as safe water, sanitation and hygiene (WASH) [5,6], mass drug administrations (MDAs), veterinary public health, vector control, and intensified case management are all critical in reducing the disease burden [3]. The MDA heads national control programs through the application of the only presently available drug, praziquantel (PZQ) with low treatment cost per individual [7,8]. While intensifying on the coverage with PZQ and therefore the drug pressure, concerns about emerging resistance should be taken into account and not taken lightly. Presence of PZQ resistance has already been recorded in experimental models [9]. The first cases of decreased drug efficacy have been observed in the laboratory-adapted isolate of *S*. *mansoni* [10] and in the field [7,11,12].

As a consequence, new drugs and alternative strategies, such as the use of vaccines, are of utmost importance to support the elimination of schistosomiasis. To support the discovery of alternatives to PZQ, *in vitro* assays of developmental stages and adult worms are crucial for decreasing dependence on *in vivo* or *ex vivo* experiments in line with the 3Rs (replacement, reduction, and refinement) of animal testing [13]. Thus, a robust, reliable and standardized way to generate advanced larval stages of *S*. *mansoni* provides an opportunity to incorporate initial advanced-stage schistosomula testing into the current drug screening cascade to meet the desired target candidate profiles (TCP), e.g. activity against juvenile stages of the schistosomes to confer chemoprevention potential.

We have recently described a novel cell-free method [14] to culture juvenile worms in a robust manner and now report improvements in the methods along with the screening of a small library of approved drugs as a usability and repurposing exercise.

## Material and methods

### Ethics statement

All animal protocols were approved and performed in accordance with the local government authorities of Bezirksregierung von Oberbayern (license no. AZ. 55.2.1.54-2532-145-17). Our animal care and use protocol are in accordance with national and European Union guidelines 2010/63.

### NCATS library

The library was a subset of the National Center for Advancing Translational Sciences (NCATS) Pharmaceutical Collection of approved and investigational drugs [15]. This subset was comprised of 340 drugs approved for human use in Europe, Canada, Japan and Australia, excluding cancer drugs. The compounds were dispensed in a 384-well plate, 30 μL at 1–20 mM concentration in dimethyl sulfoxide (DMSO) solution.

### NTS generation

Cercariae of imported NMRI strain of *S*. *mansoni* or in-house Brazilian strain were collected from infected *Biomphalaria glabrata* snails. *Biomphalaria glabrata* snails infected with the NMRI strain were provided by the NIAID Schistosomiasis Resource Center of the Biomedical Research Institute (Rockville, MD) through NIH-NIAID Contract HHSN272201700014I for distribution through BEI Resources. Screening of NCATS Pharmaceutical Collection of approved and investigational drugs and subsequent confirmation of hits was primarily performed on the NMRI strain. After collection, cercariae were mechanically transformed into NTS as described in [14]. Briefly, *cercariae* were incubated 30 min on ice followed by centrifugation at 1962 RCF for 3 min at 4°C. The supernatant was removed, and the pellet containing cercariae was resuspended in Hank’s balanced salt solution (HBSS) (Cat. No. H6648, Sigma-Aldrich, Germany) supplemented with 200 U/mL penicillin and 200 μg/mL streptomycin (Cat. No. P4333, Sigma-Aldrich). The mechanical transformation was performed through pipetting and vortexing to separate the cercarial head from tails. Tails and cercarial bodies were removed by washing three times in ice-cold HBSS supplemented with penicillin and streptomycin. After the third wash, the supernatant was removed carefully, leaving the pellet containing NTS. The pellet was resuspended in culture medium containing HybridoMed Diff 1000 (HM) (Cat. No. F 8055/1, Biochrom GmbH, Germany) supplemented with 200 U/mL penicillin, 200 μg/mL streptomycin (Sigma-Aldrich) and 20% human serum. The NTS were counted, concentration adjusted, and ~50 or ~100 NTS in 150 μL were incubated in culture medium supplemented with 20% human serum at 37°C, 5% CO_2_ for transformation to be completed.

### NTS culture

NTS (~50 or ~100 NTS in 150 μL) were grown in commercially available cell culture media HM) supplemented with 200 U/mL penicillin, and 200 μg/mL streptomycin. NTS were cultured in a 96-well flat-bottom tissue culture plate (Falcon, Reference no. 353075) and incubated at 37°C in 5% CO_2_ and humidified air for maximum 6 weeks. The medium was replaced weekly or bi-weekly to supply nutrients to support the growth of juvenile worms. For cultures intended for screening, scoring was performed on days 1, 7 post-transformation (p.t.) and then again weekly until maximum week 6 p.t. In optimization studies, an additional viability scoring timepoint at day 3 p.t. was included.

### Criteria for the viability score

The viability of NTS was scored using an Axiovert10 microscope (Zeiss, Germany) according to the scoring system described in [14]. This scoring system of NTS cultures was based on three main criteria: motility, morphology, and granularity. The score ranged from 0: dead parasites, no movement, dense granulation, blurred outline, rough outer tegument with blebs; to 1: notably reduced motility, rough outer tegument with some blebs; to 2: reduced motility or increased uncoordinated activity, intact tegument with small deformations, slight granularity; and 3: regular smooth contractions, a smooth outer surface without blebs, no granulation with a clear view of internal structures which are visible under bright field microscopy. During the mechanical transformation, individual NTS die and are, therefore, present from the beginning in culture. The number of dead parasites is taken into account when determining the viability score. An increase in the number of dead parasites will decrease the overall viability score. Viability was scored at indicated time points. Each score is an overall impression of the visual appearance of all NTS per well. Viability scores 0–3 were further subdivided into 0.25 steps (e.g. 0, 0.25, 0.50, 0.75) to capture delicate changes in the appearance of NTS.

For the determination of different developmental stages, the morphological determination was based on previously published work [14,16,17]. The skin stage (SkS) appears similar to the cercarial head in shape with undirected regular movements; the lung stage (LuS) is characterized by an increase in length and decrease in diameter. Early LiS shows clear visibility of the gut; growth of the parasite is initially observed in diameter and then in length. In the late LiS, oral and ventral suckers are recognizable, and the body length increases. The increase in body length is especially prominent past the ventral sucker.

### Growth promotion in different lots of human serum

Donor human serum (HSe) was prepared from the blood of healthy volunteers with no previous history of schistosomiasis upon written consent. Fresh blood was collected in S-Monovette^®^ Serum (Sarstedt, Cat. No. 01.1601), left at room temperature for 30–60 min to clot, then centrifuged twice at 2000 x *g* for 10 min. Serum was collected, pooled from 11 individuals and stored at -20°C until further use. Lots of commercial human serum originated from human male AB plasma, USA origin (Cat. No. H4522, Sigma-Aldrich), male AB clotted whole blood (Cat. No. H6914, Sigma-Aldrich), and from off clot pooled mixed gender, EU (Cat. No. S-106B-EU, Batch number A50618, Life Science Group).

HM supplemented with 200 U/mL penicillin and 200 μg/mL streptomycin without the addition of HSe served as a control. The medium was replenished weekly, and viability scoring was performed on day 1, 3 and 7 p.t., and then every 7 days. Developmental stages were determined by bright field microscopy using an inverted Axiovert 10 microscope (Zeiss). For each culture condition, experiments were performed at least in triplicates.

For primary screening, the culture medium contained either 20% donor or commercial (S-106B-EU) HSe. For confirmatory testing of compounds on juveniles and adult worms, commercial HSe (S-106B-EU) was supplemented in culture medium at 20%.

### Collection of adult worms

Three-week-old male NMRI mice were purchased from Envigo, Germany. Mice were infected with approximately 200–300 *S*. *mansoni* cercariae (NMRI strain) subcutaneously. After 8–9 weeks post-infection, mice were euthanized by cervical dislocation or pentobarbital. Worms were collected from the portal vein and mesenteric veins under a heated plate dissecting microscope. Adult worms were placed in a Petri dish with the culture medium on the same day after harvesting from mice, washed once, and gently transferred into 6-well plate with culture medium and left 1–2 days at 37°C, 5% CO_2_ before confirmatory testing. The culture medium for adult worms was the same as for juveniles and consisted of HybridoMed Diff 1000 (HM) (Cat. No. F 8055/1, Biochrom GmbH, Germany) supplemented with 200 U/mL penicillin and 200 μg/mL streptomycin (Sigma-Aldrich).

### Preparation of compounds for drug screening

#### Liquid compounds

For primary screening, compounds were supplied dissolved in DMSO at 10 mM and stored at -20°C until use. On the day of screening, compounds were left to equilibrate for at least 60 minutes at room temperature (RT). If screenings were performed within a month, plates with compounds were left in a desiccator at RT to avoid continuous freezing and thawing cycles.

PZQ (Cat. No. PHR1391 Sigma-Aldrich) served as a positive control, while DMSO (Cat. No. D8418 Sigma) was used as a negative control. On the day of testing, PZQ was freshly prepared by weighing and dissolving in DMSO to reach a stock concentration of 10 mM.

#### Solid compounds

For confirmatory screening on juveniles and adult worms, compounds were delivered in solid powder form and stored in the dark at RT until use. Compounds were dissolved in DMSO taking into an account the weight (g) and molecular weight of each compound to reach a stock concentration of 10 mM. The same was done for PZQ. After the addition of DMSO, vials with compounds and PZQ were vortexed at least 3x10 seconds to ensure homogeneous suspension. Reconstituted vials with compounds were kept at 5°C between experiments on juveniles and adult worms. Plate dilutions were prepared in 96-well plate for juveniles and 24-well plate for adult worms. All plate dilutions intended for primary and confirmatory screenings were performed in a laminar flow cabinet.

### *In vitro* drug testing of juveniles

On the day of testing, 21–42 day-old juveniles (depending on a study), liver stages (early and late) were scored before the addition of compounds, corresponding to time point 0 hours before drug treatment. Each well had to contain at least 3–4 late liver stages to be selected for screening. In the case of NMRI strain, the majority of juveniles were in an early liver stage. After selection of wells for screening, 96-well plate containing juveniles were washed 3x with HM supplemented with 200 U/mL penicillin and 200 μg/mL streptomycin to remove metabolites and waste generated during cultivation. For primary screening, preparation of compounds and controls was performed in two 96-well plates. In plate 1, compounds and PZQ were diluted to 100 μM in distilled water, and further diluted in HM supplemented with 200 U/mL penicillin, 200 μg/mL streptomycin and 20% HSe (hereafter termed culture medium) in plate 2. After juveniles were washed 3x with HM supplemented with 200 U/mL penicillin and 200 μg/mL streptomycin, the supernatant was removed, and 150 μL of plate 2 was transferred to wells with worms (plate 3). The final concentration of tested compounds and PZQ was 30 μM. DMSO was tested at 0.3% v/v (volume by volume; representative of the highest solvent concentration). Compounds were tested in single replicates and controls in duplicates.

For confirmatory screening, compounds and PZQ were diluted in distilled water to 100 μM in plate 1. From plate 1, compounds and PZQ were further diluted in the culture medium on plate 2 to three concentrations: 3.333 μM, 10 μM, and 30 μM. The final concentration of DMSO on plate 2 was 0.3% v/v. As in the case of primary screening, wells containing juvenile worms (plate 3) were washed 3x in HM supplemented with 200 U/mL penicillin and 200 μg/mL streptomycin, after which, the supernatant was removed, and 150 μL of plate 2 was transferred to wells with worms. DMSO was tested at 0.3% v/v (representative of the highest solvent concentration). Compounds, PZQ, and DMSO, were tested in duplicates.

For primary and confirmatory screening, after the addition of compounds, worms were incubated at 37°C, 5% CO_2_, and scored after 3 hours (h), 24 hours, and day 7 post-treatment using an inverted Axiovert 10 microscope (Zeiss) at 10x magnification.

Primary screening was performed using *S*. *mansoni*, NMRI strain, except in one study (Thioproperazine in Table 1). In this study, thioproperazine screened as a hit on liver stages of *S*. *mansoni*, Brazilian strain. Confirmation of primary hits was always carried out with the NMRI strain. As the majority of schistosomula in each well were in liver stage for NMRI strain, and the aim was to assess the activity of compounds on juveniles, viability score was assigned only to liver stages. Consequently, during primary screening and confirmation, hits were determined if the viability score of liver stage (early and late) was ≤ 1.25 at 24 h after exposure to compounds. Thus, the effect of compounds on lung stages was not considered when assigning a viability score. All information on the phenotype of lung stages in Table 1 is for information only.

**Table 1:** List of primary hits.

Compound Structure & Name	CLASS	MW g/mol	LogP	TPSA Å^2^	Primary screening		Confirmation of hits	
					Viability score juveniles 24h incubation (n = 1) 30 μM	Observed phenotype at primary screening (30 μM), juvenile *S*. *mansoni*	Viability score juveniles at 24h (n = 2) Dose response	Viability score adults at 24h (n = 2) 30 μM
**DMSO**					2.35 ^A)^	Liver stage (LiS) and lung stage (LuS): Active, shape and form as expected. No specific changes to 0h and 3h.	0.3% v/v: 2.31^B)^	2.75
**Praziquantel**	ANTIPSYCHOTIC				0.50 ^A)^	LiS: Phenotype is as seen at 3h. LiS are granulated, contracted with heavily reduced motility (only of the anterior part). A few appear disintegrated. LuS: Pearl-like, contracted with heavily reduced activity (only of the anterior part).	30 μM: 0.50 ^C)^ 10 μM: 0.64 ^C)^ 3.333 μM: 0.77 ^C)^	0.25
Thioproperazine ^1)^		446.6	4.3	80.8	1.00	Lung stage (LuS): Sluggish, damaged inner structure and some are granulated. Liver stage (LiS): Heavily granulated and darker brown. Still active movements, but reduced compared to 3h.	30 μM: 1.00, 0.75 10 μM: 1.00, 1.25 3.333 μM: 1.75, 1.75	1.75, 2.00
cis-Flupentixol		434.5	5.1	52.0	1.25	LiS: Reduced activity; increased granularity than at 3h. A few appear damaged inside or disintegrated. LuS: Reduced activity, surface damaged.	30 μM: 1.25, 1.25 10 μM: 1.25, 1.50 3.333 μM: 1.75, 1.75	2.00, 1.75
Spiperone		395.5	2.9	52.7	1.25	LuS appear slightly damaged but otherwise unaffected. LiS: Increased granularity in LiS and some are deformed or damaged. Activity is present but reduced.	30 μM: 1.00, 1.00 10 μM: 1.50, 1.25 3.333 μM: 1.75, 1.50	2.25, 2.50
Sertindole		440.9	3.5	40.5	1.25	LuS: Reduced activity, surface damaged. LiS: Overall activity is present, there is an increase in granularity compared to 3h (darker brown inside), and in some, it is more pronounced. A few are deformed or damaged.	30 μM: 1.00, 0.75 10 μM: 1.25, 1.25 3.333 μM: 1.50, 1.50	2.00, 2.25
Nefopam	ANTINOCEPTIVE	253.3	3.2	12.5	1.25	LuS: Same as 3h (Activity is reduced than at 0h; minor granularity). LiS: Some are with damaged internal structures. A few are heavily granulated or deformed in shape.	30 μM: 1.50, 1.25 10 μM: 1.75, 1.75 3.333 μM: 1.75, 1.75	2.50, 2.50
Bifemelane	ANTIDEPRESSANT	269.4	3.9	21.3	1.25	LuS: Activity not as hectic as in LiS; surface damaged. LiS: Hectic movements, increased granularity (in some more than the others), the gut is dark brown.	30 μM: 1.00, 1.25 10 μM: 1.25, 1.50 3.333 μM: 1.75, 1.50	2.25, 2.00
Dibenzepin ^2)^		295.4	2.6	26.8	1.25	LuS: Activity is present, slightly surface damaged. LiS: Increased granularity compared to 3h, bubbles are still present, some are deformed. Activity is overall present but somewhat reduced.	Not tested	Not tested
Pizotyline		295.4	4.6	31.5	1.25	LuS: LuS are with increased granulation than at 3h. Overall the activity is present but reduced. LiS: Increased granularity in the majority of LiS. Some are deformed in shape.	30 μM: 1.75, 1.50 10 μM: 1.75, 1.75 3.333 μM: 2.00, 1.75	2.75, 2.50
Ifenprodil	CNS	325.4	3.1	43.7	1.25	LuS: Reduced activity, surface damaged. LiS: Activity is overall reduced, increased granularity compared to 3h. A few appear damaged (disintegration has started).	30 μM: 1.00, 1.00 10 μM: 1.25, 1.50 3.333 μM: 1.75, 1.50	2.50, 2.25
Fasoracetam ^2)^	NOOTROPIC				1.25	LuS: No specific changes. LiS: Granularity is more evident than at 3 h, a few are damaged, and activity is reduced.	Not tested	Not tested
Paraoxon ^2)^	CHOLINERGIC				1.25	LuS: There are no specific changes. LiS: Activity is present. Granularity is increased compared to 3 h, and worms have bubbles on their surface. A few are deformed or damaged (disintegrated).	Not tested	Not tested
Aprindine	CARDIOVASCULAR	322.5	4.5	6.5	0.25	LuS: Heavily granulated and do not seem to move (flickering-like as in LiS). LiS: All are densely granulated, and internal structures cannot be seen. In some two brown spots are visible inside, and some are like disintegrating inside. Some are showing disintegration, i.e. rough tegument. They do not appear move but appear paralyzed (flickering-like of internal structures).	30 μM: 1.00, 1.25 10 μM: 1.50, 1.25 3.333 μM: 1.75, 1.75	2.25, 2.50
Perhexiline		277.5	4.5	12.0	0.75	Liver stages are heavily granulated, and some are deformed. Movements are irregular and only of the anterior part (in most liver stages). LuS with slight irregular movements, and granulation.	30 μM: 0.75, 0.50 10 μM: 1.50, 1.25 3.333 μM: 1.75, 1.50	1.00, 1.25
Gallopamil ^3)^		484.6	4.6	73.2	1.25	LuS: Reduced activity, appear surface damaged. LiS: More granulated than at 3h, some are darker than the others. Activity is reduced. A few are deformed or damaged or disintegrated.	30 μM: 1.00, 1.25 10 μM: 1.25, 1.25 3.333 μM: 1.25, 1.50	Run 1: 2.25, 2.25, Run 2: 2.50, 2.25
Dilazep	VASODILATOR	604.7	3.5	114.5	1.25	LuS: Reduced activity; surface damaged. LiS: Increased granularity. Some are bloated, deformed or damaged inside. The overall movement is present but reduced.	30 μM: 1.75, 1.75 10 μM: 1.75, 2.00 3.333 μM: 2.00, 2.00	2.00, 1.75
Astemizole ^3)^	ANTI-ALLERGIC	358.6	4.9	42.3	1.00	LuS: Reduced motility, surface damaged (like vacuole-inside) or densely granulated. LiS: Densely granulated (darker in colour) compared to 3 h. Motility is decreased. Some are deformed.	30 μM: 0.75, 1.00 10 μM: 1.25, 1.50 3.333 μM: 2.00, 1.75	Run 1: 1.00, 1.25, Run 2: 1.25, 1.00
Alverine	ANTISPASMODIC	281.4	4.1	3.2	1.25	LuS: Reduced activity; surface damaged. LiS: Increased granularity compared to 3 h, some are deformed in shape. The activity is present but overall reduced.	30 μM: 1.00, 1.25 10 μM: 1.25, 1.25 3.333 μM: 1.50, 1.75	2.25, 2.00
Camylofin		320.5	2.9	41.6	1.25	LuS: Activity is present with slight granularity or a few are damaged inside. LiS: Increased granulation in some LiS. Some are deformed in shape. Activity is present. A few have a big vacuole-like inside (damaged).	30 μM: 1.50, 1.25 10 μM: 1.75, 1.50 3.333 μM: 2.00,1.75	2.75, 2.50
Berberine	ANTI-INFECTIVE	371.8	-1.3	40.8	0.75	LuS: Activity is present but appears surface damaged. LiS: Contracted, round-shaped with increased granularity. Movement is only of the anterior part. A few are disintegrated.	30 μM: 1.25, 1.25 10 μM: 1.50, 1.50 3.333 μM: 2.00, 1.75	2.25, 2.25
Sanguinarine ^2)^		-	-	-	0.25	LuS: Same LiS and some have a vacuole visible inside, like the disintegration of internal structures. LiS: All are densely granulated (dark brown), the internal structure is not clear; rough tegument as some are disintegrating. The phenotype is very similar to G05. No visible movement; overall it appears like the flickering of the tegument.	Not tested	Not tested

Abbreviations: CNS: Central nervous system; DMSO: Dimethyl sulfoxide; h: hours; LiS: liver stage; LuS: lung stage; v/v: volume by volume

Mean for

^A)^ n = 38,

^B)^ n = 86,

^C)^ n = 16

^1)^ Thioproperazine: Primary screening was based on using the Brazilian strain. In confirmation experiments, the NMRI strain was used both for juveniles and adult worms.

^2)^ Dibenzepin, Fasoracetam, and Sanguinarine were tested during primary screening. The compounds were not tested during the confirmation phase.

^3)^ Gallopamil and Astemizole were tested twice (run 1 and run 2) in duplicates.

### *In vitro* drug testing of adult worms

On the day of screening, plates with adult worms were inspected under an inverted Axiovert 10 microscope (Zeiss) at 5x magnification. Gently using forceps, male and female worms transferred into 24-well plate containing the culture medium. Each well included at least 3 male and 3 female worms. More specifically, each well either contained individual worms or 2 male and 2 female as single worms and 1 pair. After the selection of adult worms, plates with worms and culture medium were placed in a laminar flow cabinet.

In plate 1, compounds and PZQ were diluted in distilled water to 100 μM. From plate 1, 450 μL was transferred to plate 2 already containing adults worms in culture medium to a final volume 1500 μL. Compounds and PZQ were screened at 30 μM, and DMSO at 0.3% v/v (representative of the highest solvent concentration). Compounds, PZQ, and DMSO, were tested in duplicates. After the addition of compounds, plates with worms were placed at 37°C, 5% CO_2_. Viability scoring was performed after 3, 24, and 72 h post-treatment under an inverted Axiovert 10 microscope (Zeiss) at 5x and 10x magnification. Hits were confirmed if the average viability score was ≤ 1.25 at 24 h after exposure to compounds. After a 24-hour exposure, compounds and controls were washed 3x in HM supplemented with 200 U/mL penicillin and 200 μg/mL streptomycin, after which adult worms were then incubated in culture medium only.

### Drug wash-out after a 24-hour incubation time

To monitor the effect of compounds on worm cultures, i.e. reversible or irreversible, compounds and controls were removed after a 24-hour exposure from juvenile and adult worm cultures during primary and confirmatory screenings. The supernatant containing compounds was discarded, and worms were washed 3x in HM supplemented with 200 U/mL penicillin and 200 μg/mL streptomycin. After the third wash, the supernatant was removed, and the culture medium was added to each well. Plates were returned to the incubator at 37°C, 5% CO_2_. The final viability score as a measure of recovery was done on day 7 and day 3 from the initial screening for juvenile and adult worms, respectively.

### Scanning electron microscopy (SEM)

*S*. *mansoni* juveniles were cultured in the culture medium containing commercial human serum (S-106B-EU) until day 29. Juveniles were collected in protein low binding Eppendorf tube and centrifuged for 10 min at 5000 x g at 22°C. The supernatant containing the culture medium was discarded. Samples were treated to series of sequential steps: Fixation (4% glutaraldehyde and 0.2 M cacodylate), 1^st^ wash (0.4 M saccharose / 0.2 M cacodylate), post-fixation (2% osmic acid / 0.3 M cacodylate), 2^nd^ wash (distilled water) followed by ethanol dehydration at 50%, 70%, 90%, and 100% concentration. Samples were then air-dried, mounted, and coated with gold sputter before imaging.

### Transmission electron microscopy (TEM)

Schistosomula 29 days (7 weeks) and 77 days (11 weeks) old were fixed in buffered 2.5% Glutaraldehyde phosphate (0.2 mol/L, pH 7.4) in 0.1 M Sodium cacodylate. Post-fixation was carried out in cold buffered 1% Osmium tetraoxide, followed by dehydration in ascending gradients of ethanol (50%, 70%, 90%, 100%) before embedding in Epoxy resin. Samples were embedded in Epon (Serva Electrophoresis GmbH). 60–70 nm thick ultrathin sections were cut at the Reichert-Jung Ultracut E microtome (Darmstadt, Germany) microtome. Ultrathin sections were collected on formvar coated copper grids (Plano, Germany) and automatically stained with UranyLess EM Stain (Electron Microscopy Sciences) and 3% lead citrate (Leica, Wetzlar, Germany) using the contrasting system Leica EM AC20 (Leica, Wetzlar, Germany). Imaging was carried out using the JEOL -1200 EXII transmission electron microscope (JEOL, Akishima, Tokyo) at 80 kV. Images were taken from the dorsal surface of the worm using a digital camera (KeenViewII; Olympus, Germany) and processed with the iTEM software package (anlySISFive; Olympus, Germany).

### Computational chemistry

Physiochemical properties of compounds were calculated using the CDK package in KNIME Analytics Platform v3.6.1 [18]. Statistical significance of differences between properties of active and inactive compounds was measured by the Student’s t-test p-value. Three properties were found significantly different. XLogP is a measure of the octanol/water partition coefficient. Compounds with larger XLogP values are more lipophilic. Bond polarizability is the ability of a bond to form instantaneous dipoles. Polarizabilities determine the dynamical response of a bound system to external fields and provide insight into a molecule’s internal structure. Fragment complexity is a measure of the structural complexity of a molecule.

### Calculation of Z’- factor and SSMD

Z’-factor is a statistical parameter used for quality assessment during assay development and optimization, which takes into consideration positive and negative assay controls without the interference of test compounds. Z’-factor values of 0.5–1 indicate excellent assay performance. Calculation of Z’-factor was done as described in [19] as follows:

$$Z^{\prime }=1-\frac{\left(3\sigma_{\text{c}+}+3\sigma_{\text{c}-}\right)}{\left|\mu_{\text{c}+}-\mu_{\text{c}-}\right|}$$

Where,

σ_c+_ is the standard deviation of positive control

σ_c-_ is the standard deviation of negative control

μ _c+_ is the mean of positive control

μ _c-_ is the mean of negative control

Another, more robust, statistical parameter to evaluate assay performance is the strictly standardized mean difference (SSMD). The recommended threshold as an indicator of data quality is SSMD of 6 [20].

Calculation of SSMD was performed as described in [20]:

$$SSMD=\mu_{\text{h.c}.}-\mu_{\text{l.c}}\text{/}\sqrt{{\sigma^{\text{2}}}_{\text{h.c}}+{\sigma^{\text{2}}}_{\text{l.c}}}$$

Where,

σ _h.c_. is the standard deviation of high control (signal from negative control, DMSO)

σ _l.c_. is the standard deviation of low control (signal from positive control, PZQ)

μ _h.c_. is the mean of high control (signal from negative control, DMSO)

μ _l.c_. is the mean of low control (signal from positive control, PZQ)

### Statistical analysis

Statistical analysis and preparation of graphs were done using PRISM 8 (GraphPad). Data were tested for normality in PRISM 8. One-way ANOVA using the non-parametric Kruskal-Wallis test, followed by Dunn’s multiple comparison test was used to compare multiple test groups. Comparison between two groups was performed with the non-parametric Mann-Whitney test (Compare ranks). In all cases, p ≤ 0.05 was considered significant.

## Results

### Part 1: Assay optimization for drug screening

#### Growth of juveniles under *in vitro* conditions is dependent on serum lot

**Fig 1:**
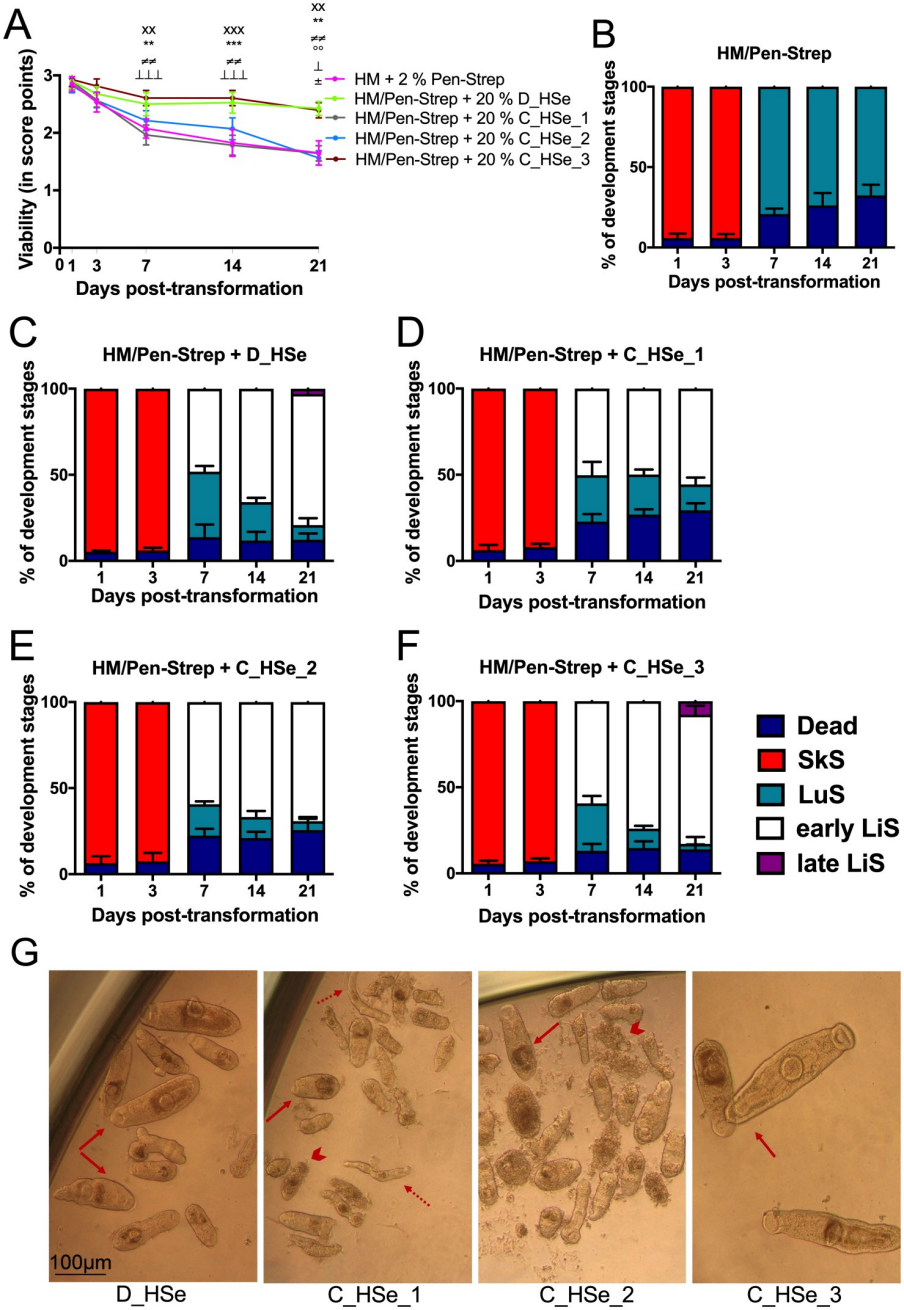
Development of *Schisotoma mansoni* juveniles in presence of human serum from different sources. Juveniles were cultured in HM supplemented with 200 U/mL penicillin and 200 μg/mL streptomycin as well as 20% D_HSe (serum from human donors), 20% C_HSe_1 (Commercial human serum from human male AB plasma, H4522), 20% C_HSe_2 (Commercial human serum from male AB clotted whole blood, H6914), and 20% C_HSe_3 (Commercial human serum, pooled, mixed gender, Off clot, S-106B-EU). (A) Viability was scored during bright field microscopy. The percentages of the developmental stages as well as dead parasites in culture with (B) Hybridomed Diff 1000 supplemented with 200 U/mL penicillin and 200 μg/mL streptomycin (HM/Pen-Strep) or also supplemented with (C) 20% D_HSe, (D) 20% CHSe_1, (E) 20% C_HSe_2, and (F) 20% CHSe_3 at indicated time points and (G) Representative photomicrographs were taken on day 21 post-transformation at 10x magnification. In photomicrographs: broken arrow (lung stage), arrow (liver stage), and arrowhead (dead schistosomula). Results for the percentages of the developmental stages are based on four replicates per condition per time point. Viability results are pooled from three independent experiments with at least three biological replicates each. ^xx^p ≤ 0.01, ^xxx^p ≤ 0.001 comparing D_HSe vs HM; **p ≤ 0.01, ***p ≤ 0.001 comparing D_HSe vs C_HSe_1; ^≠≠^p≤0.01 comparing D_HSe vs C_HSe_1; ^⊥^p ≤ 0.05, ^⊥⊥⊥^p ≤ 0.0001 comparing C_HSe_1 vs C_HSe_3; °°p ≤ 0.01 D_HSe vs C_HSe_2; ^±^p ≤ 0.05 comparing CHSe_2 vs C_HSe_3 by Kruskal-Wallis non-parametric test followed by Dunn’s multiple comparison tests. C: Commercial; D: Donor; HSe: Human serum; HM: Hybridomed Diff 1000; Pen/Strep: Penicillin-streptomycin; SkS: Skin stage; LuS: Lung stage; LiS: Liver stage.

Our recently developed *in vitro* culture system to generate juvenile worms depends on a reliable source of human serum which up to now was an in-house serum pool derived from individual donors. To standardize the procedure, different lots of commercial human serum showed pronounced differences in the development of worms. *Schistosomula* incubated in commercial human serum, C_HSe_1 (H4522) and C_HSe_2 (H6914) and the culture medium alone, showed steady overtime decline in viability score (Fig 1A). In HM supplemented with 200 U/mL penicillin and 200 μg/mL streptomycin (HM/Pen-Strep), worms did not develop beyond the lung stage (Fig 1B). Already at day 7, the viability of schistosomula in C_HSe_1 was significantly lower compared to the ones incubated in donor serum (D_HSe), p = 0.0018 and commercial serum from EU origin (S-106B-EU, C_HSe_3), p = 0.0004. This significant downward trend in the viability of schistosomula grown in C_HSe_1 continued at day 14 and day 21 (Fig 1A). At day 21, schistosomula cultured in C_HSe_2 had a viability score 1.6 ± 0.1, and significantly lower than in D_HSe (2.4 ± 0.1, p = 0.0054) and C_HSe_3 (2.4 ± 0.1, p = 0.0190), Fig 1A. The impaired growth in C_HSe_1 and C_HSe_2 was confirmed with development halting at the early LiS at day 21 (Fig 1D and 1E). As shown by images taken at day 21, the early LiS appeared stunted in growth in the presence of deformed and dead parasites, Fig 1G. Contrary to C_HSe_1 and C_HSe_2, incubation of schistosomula in C_HSe_3 (Fig 1F) promoted worm growth. Overtime viability score stayed comparable between D_HSe and C_HSe_3. Similarly, development at days 1–21 followed the similar trend, however, with a higher percentage of late LiS in C_HSe_3 (Fig 1F) compared to D_HSe (Fig 1C) (3 ± 1% versus 8 ± 3%, respectively; mean ± standard deviation). As seen in Fig 1G, juvenile worms incubated in C_HSe_3 developed to early and late LiS with a shaped oral and ventral sucker, clearly outlined bifurcated gut, and intact body at day 21, as seen under bright field microscopy. Similar observations in shape and form of LiS were seen when incubated in D_HSe (Fig 1G): However, these advanced LiS were visually smaller when compared to C_HSe_3, so were those raised in commercial serum originating from the US donors (C_HSe_1 and C_HSe_2). This was especially observed in the case of C_HSe_2, whereby worms appeared disintegrated with some LiS darker in colour and heavily granulated (Fig 1G).

From Fig 1, commercial human serum, code S-106B-EU (pooled, mixed gender, off clot) showed comparable performance in the viability of the parasites and development of liver stages (early and late) compared to serum from donors. This allows reproducibility and sustained access to similar batches. Therefore, all following cultivation experiments were performed using commercial human serum S-106B-EU (C_HSe_3).

**Fig 2:**
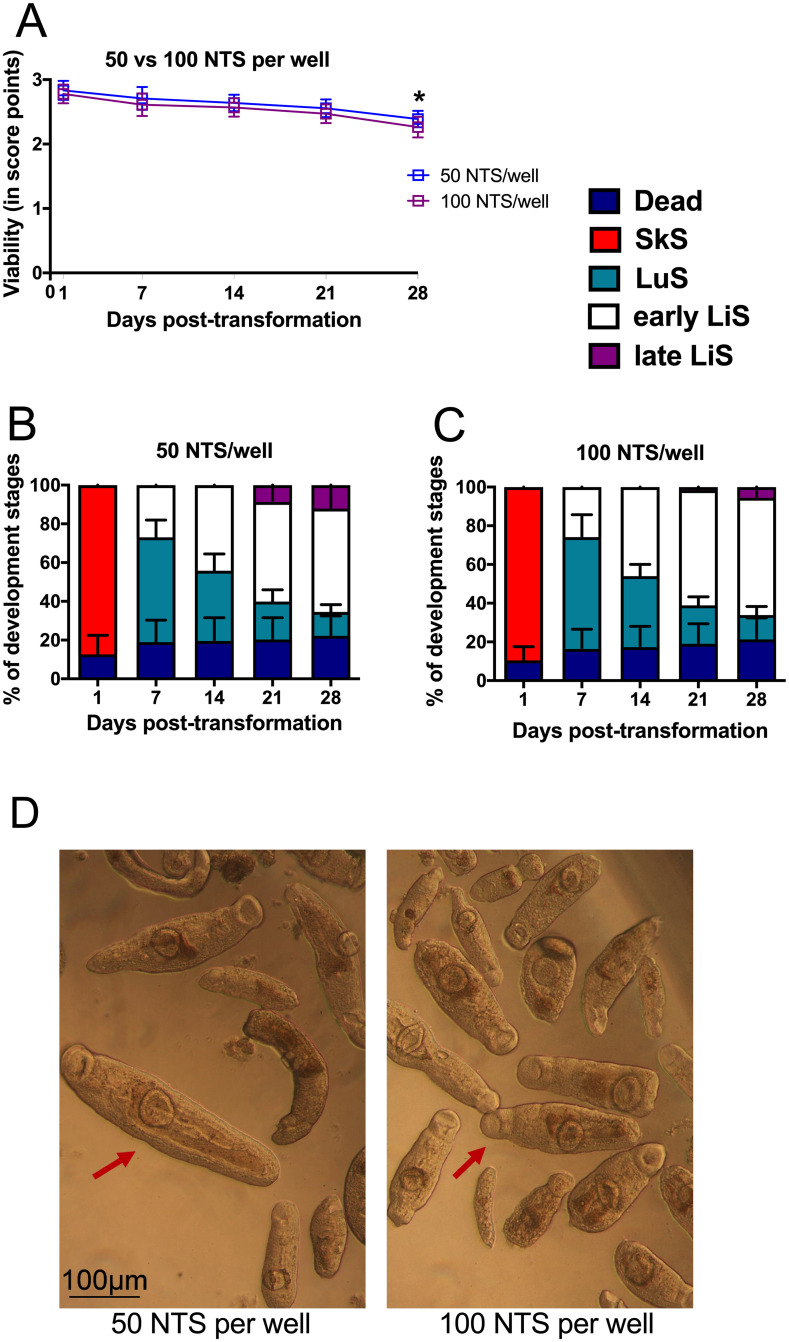
Downscaling the number of NTS. Approximately 50 and 100 NTS were cultured in a 96-well format with HM supplemented with 200 U/mL penicillin, 200 μg/mL streptomycin and 20% human serum (A) Viability was scored during bright field microscopy. The percentages of the developmental stages as well as dead parasites in culture with (B) 50 NTS/well and (C) 100 NTS/well. (D) Representative photomicrographs were taken on day 28 post-transformation at 10x magnification. Arrowheads indicate the liver stage. Data represents mean ± SD for n = 18 per time point. *p ≤ 0.05 comparing 50 NTS vs 100 NTS per well at day 28 post-transformation by Mann-Whitney non-parametric test. NTS: Newly transformed schistosomula; HM: Hybridomed Diff 1000; SkS: Skin stage; LuS: Lung stage; LiS: Liver stage.

Next, we aimed at optimizing the assay by evaluating whether a decrease in NTS from 100 to 50 per well would either maintain or increase the viability and the rate of development. As shown in Fig 2A, viability score had a similar trend among the two groups. At day 28, viability score was significantly higher in the group with the initial approximately 50 NTS per well (p = 0.0288). As shown in Fig 2B and 2C, similar developmental patterns were observed in the “50 NTS” and “100 NTS” group. The majority of worms developed from schistosomula (day 1) to LuS on day 7. From day 7 onwards, early LiS started developing, and their number increased steadily in both groups from day 14 to day 28 (Fig 2B and 2C). However, late LiS developed in higher numbers in the 50 NTS group at day 21 (9% ± 5% in 50 NTS *versus* 2 ± 2% in 100 NTS group) and day 28 (12 ± 3% in 50 NTS versus 5 ± 3% in 100 NTS group), respectively. Furthermore, individual late liver stages in the 50 NTS group appeared bigger compared to the 100 NTS group, as seen in Fig 2D.

Taken together, these results show that decreasing the number of NTS from 100 to 50 gave a steady viability score at different time points and a higher percentage of late LiS. Consequently, decreasing the initial NTS per well increased the output of screened compounds, and further optimized the assay.

**Fig 3:**
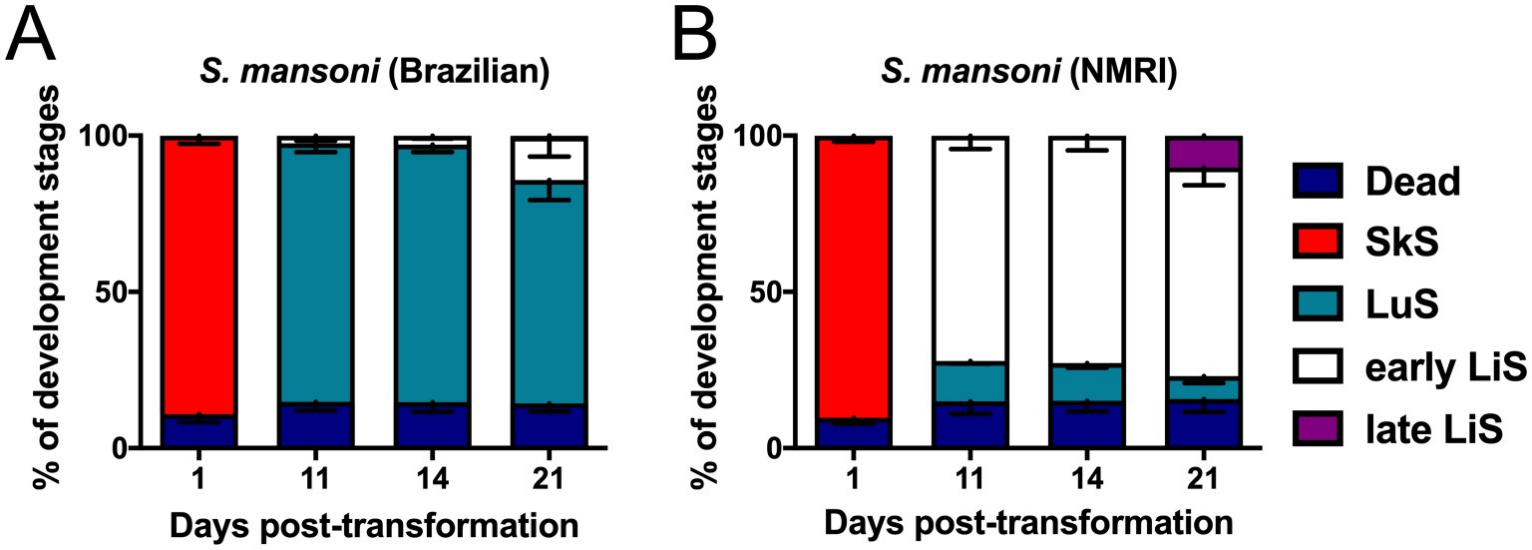
Differences in the development of *Schistosoma mansoni* strains. Juveniles were cultured in HM supplemented with 200 U/mL penicillin and 200 μg/mL streptomycin and 20% commercial human serum (S106B-EU). The percentages of the developmental stages, as well as dead parasites, were assessed in culture with (A) *S*. *mansoni* Brazilian strain and (B) *S*. *mansoni* NMRI strain. Results for the percentages of the developmental stages are based on five replicates per condition per time point. HM: Hybridomed Diff 1000; SkS: Skin stage; LuS: Lung stage; LiS: Liver stage.

Further, we investigated whether there is a strain-specific development when subjecting *S*. *mansoni* Brazilian and NMRI strains to the same *in vitro* culture conditions. Percent of development was monitored over three weeks. As shown in Fig 3, the Brazilian (Fig 3A) and NMRI (Fig 3B) worms showed a clear difference in the pace of development. While both groups were in schistosomula stage at day 1, at day 11, the majority of parasites remained as LuS in the Brazilian strain (Fig 3A), with 14 ± 6% early LiS and 0.4 ± 0.8% late LiS developed at day 21 (Fig 3A). Contrary to slow pace development of the Brazilian strain, the NMRI strain developed faster. At day 11, the majority of worms were in early LiS (72 ± 4%) and remained the main larval stage until day 21 (Fig 3B). By day 21, 10 ± 2% NMRI worms have grown to late LiS (Fig 3B). Over time development of the NMRI strain from day 1 to 21 is presented in S1 Fig. As shown in S1 Fig, by day 7, schistosomula developed to lung stage and early liver stage in 20% human serum. An increase in early liver stages was observed at day 14 and 21. Late liver stages were seen on day 21 (S1 Fig). Although the NMRI worms developed faster, their viability significantly decreased from day 21 onwards (S2 Fig). Thus, at day 21, viability at day 21 was significantly higher compared to viability at days 28, 35, and 42 (p < 0.0001).

Furthermore, the lowest viability was recorded at day 42 (p < 0.0001 compared to day 28 and p = 0.0005 compared to day 35). Data, therefore, shows that screening of worms is recommended at peak viability, i.e., day 21. Taken together, our results clearly demonstrate that the choice of parasite strain and their inherent pace of development is an important consideration when choosing the time point of the compound screening. Within the same *S*. *mansoni* strain, i.e., NMRI or Brazilian strain, time of testing is very much dependent on the right balance between the development to late liver stages and peak viability.

The electron microscopic examination of the 29–day old schistosomula tegument (NMRI strain) from its dorsal side showed a granular cytoplasm containing scattered small mitochondria and many inclusion bodies that differ in size and shape (S3A–S3C Fig). Some have a large central electron-dense core and a lucid periphery. Invaginations in the tegument can be seen giving the tegument a sponge-like appearance. Erupting spines that face posteriorly are featured in the tegument (S3C Fig). A basal membrane separates the tegument from the underlying body parenchyma. Directly beneath the basal membrane, an external layer of circular muscle is seen situated above an internal layer of longitudinal muscle. Myocytes forming the body wall musculature of the schistosomula are seen embedded and surrounded by the parenchyma and the basal layer of the tegument. They extend in long, tortuous processes (myofibers) throughout the schistosomula. In their densely granular sarcoplasm lie the large, irregularly-shaped and heterochromatic nucleoli. Size analyses via SEM revealed schistosomula with a length of about 0.75 mm, round in shape and with clearly visible and well-developed oral and ventral suckers. Their tegument shows ruffle-like folds with each fold showing outgrowths and indentations (S5A–S5E Fig).

TEM of the 77–day old parasite tegument shows the exact characteristics of the 29–day old schistosomula described above except that the spines are larger in size and more in number (S4A–S4C Fig). Abnormal or damaged mitochondria with edema and loss of cristae are depicted in S4C Fig. Taken together, the results by TEM show that advanced development under these *in vitro* conditions can already be reached by day 29.

### Part 2: Screening of compounds

**Fig 4:**
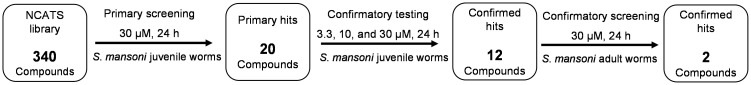
Description of the screening cascade.

Screening of compounds was performed in several stages, as shown in Fig 4. The first phase, “primary screening” was performed on 340 compounds from NCATS library. In vitro cultured juvenile worms were screened in singlets at the concentration 30 μM. Hits were determined if the viability score was ≤ 1.25 at 24 h before compounds were washed by three changes of HM medium supplemented with 200 U/mL penicillin and 200 μg/mL streptomycin. Afterwards, worms were incubated in the culture medium only. Out of 340 compounds, 20 compounds were identified as primary hits. In the next step, these hits were then tested on *in vitro* juvenile worms at three concentrations (3.333 μM, 10 μM, and 30 μM) in duplicates. The same compounds were also tested on adult worms at a single concentration, 30 μM. In both juveniles and adult worms, hits were determined if the average viability score per concentration was ≤ 1.25. From 20 primary hits, 12 compounds were confirmed on juveniles and 2 on adult worms (Fig 4). Details on hits, viability scores, and the description of phenotypes on juveniles are presented in Table 1 and S8 Fig. A list of compounds not classified as primary hits is provided in S2 Table.

**Fig 5:**
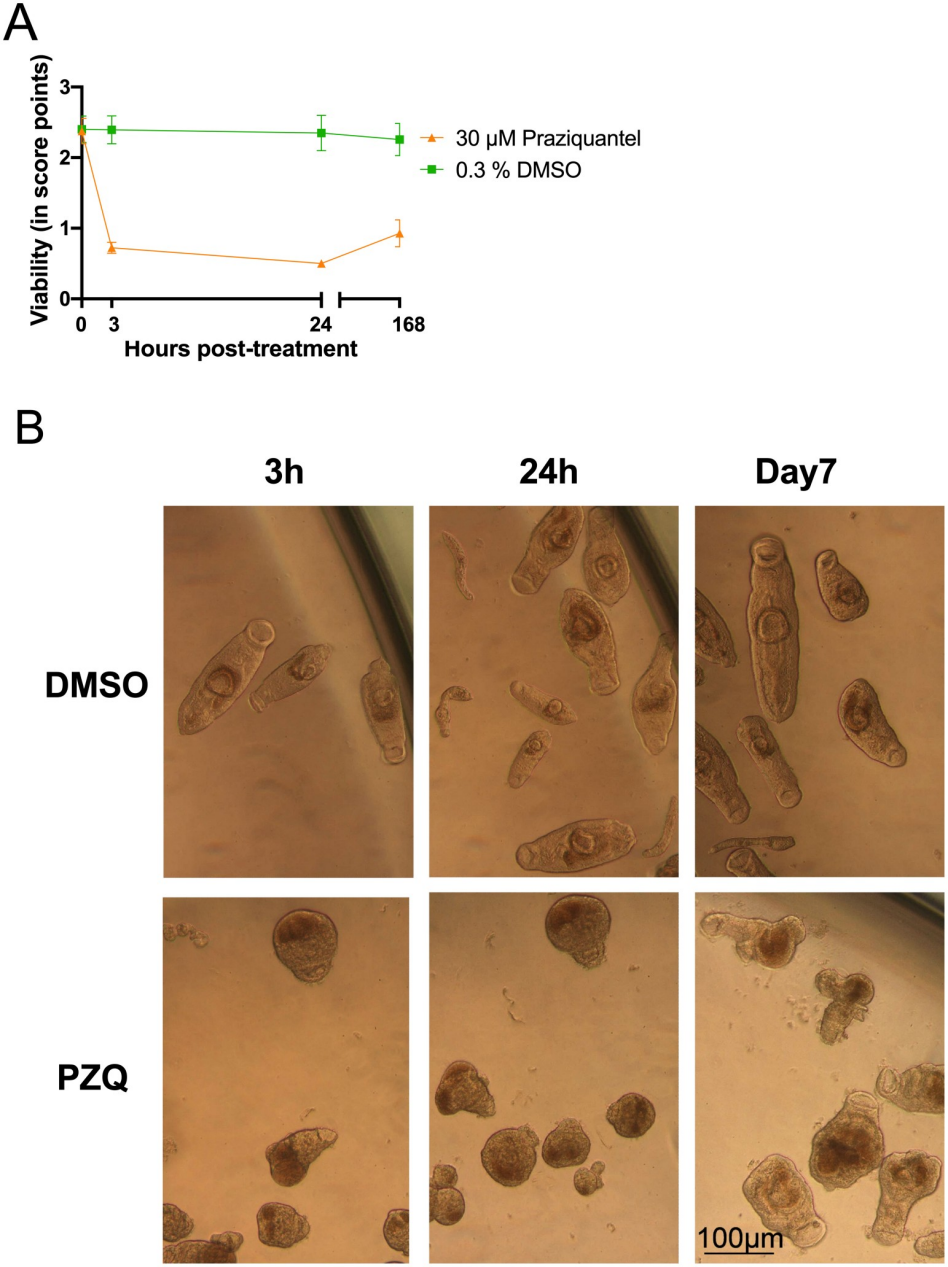
Assay controls for primary screening. A) Viability scoring of *in vitro S*. *mansoni* juvenile cultures (day 21, day 28, and day 42). During assay optimization of the drug screening protocol, the time of screening was decreased from day 42 to day 28, and finally to day 21 for the NMRI strain. All, except one study during primary screening, were performed using the NMRI strain. One study was conducted using the Brazilian strain. Positive control (Praziquantel, PZQ) was added at 30 μM. Dimethyl sulfoxide (DMSO) served as a negative control at 0.3% v/v. Juveniles were scored before the addition of controls (= 0 hours), and 3 h and 24 h post-treatment (p.t). After 24 h incubation, controls were removed from wells and replaced with culture medium (Hybridoma Diff 1000 supplemented with 200 U/mL penicillin, and 200 μg/mL streptomycin and 20% human serum) without PZQ or DMSO. Recovery of juveniles was assessed on day 7 (168 h) from the initial drug screening. Data represent the mean ± standard deviation for n = 38 per time point for a total of B) The representative phenotype of juveniles treated with 30 μM PZQ and 0.3% v/v DMSO. 10x magnification; h: hours; v/v: volume by volume.

In the establishment of primary screening platform, two controls were selected. PZQ at 30 μM served as a positive control and DMSO at 0.3% v/v (representing the highest concentration of solvent). Assay performance was monitored by the inclusion of positive and negative controls on each plate and applying the same conditions as for compounds, i.e., PZQ and DMSO were added to cultures and viability scoring was monitored at 3 h and 24 h p.t (Fig 5). The results are pooled from a total of 18 runs (each run was performed in duplicate or quadruplicate). After 24 h and removal of controls, recovery of worms was checked at day 7 from the initial addition of controls. As shown in Fig 5A, PZQ already at 3 h p.t. had an effect on juveniles with decreased viability below 1.00. The effect decreased further to 0.50 after 24 h (Fig 5A). Although strongly affected by PZQ, juveniles were still alive, and after removal of PZQ, they recovered to some extent at day 7 (168 h) (Fig 5A). Under bright field microscopy, juveniles appeared contracted, darker in color, heavily granulated (Fig 5B). Motility was severely affected with an only anterior part slightly moving. Even in the presence of the highest solvent concentration, juveniles did not appear affected by DMSO at 0.3% v/v (Fig 5A), and viability score remained unaffected at 3 h and 24 h p.t. At day 7 (168 h), the average viability score slightly decreased from 2.35 ± 0.25 to 2.26 ± 0.23, but not significantly (p = 0.0772). Under bright field microscopy, no apparent changes in phenotype and morphology of worms were seen at 3 h, 24 h, and day 7 (168 h) p.t. (Fig 5B) in the negative control; clear body outline, visual oral and ventral suckers, and bifurcated gut was observed at all times (Fig 5B). Overall, motility in the DMSO group was not affected. Taken together, the results have shown a consistent performance of positive (PZQ) and negative (DMSO) controls over time.

Z’ and SSMD were calculated for each screening time point (0 h, 3 h, 24 h, and day 7) for juvenile and adult worms. As shown in S1 Table, Z’ ranged from 0.4–0.8 and SSMD between 7.0–19.6, depending on the parasite stage and PZQ concentration. The highest Z’ and SSMD at 3h was calculated for adult worms. Similarly, the highest Z’ at 24 h was calculated for adult worms. Among juveniles, SSMD and Z’ were comparable among three different concentrations of positive control (3.333 μM, 10 μM, and 30 μM).

At day 7, after washout of positive and negative control at 24 h, both Z’ and SSMD decreased. The downward trend was observed regardless of parasite stage (juveniles or adult worms), thus, ranging from 0.1–0.5 for Z’ and 4.9–7.9 for SSMD. These results point to 3 h, and 24 h are the most relevant from Z’ and SSMD standpoint where PZQ decreased viability and produced the phenotype. However, after the washout, our results showed that PZQ is unable to sustain its activity, consequently resulting in poor Z’ and SSMD.

Out of 20 compounds confirmed as primary hits, 12 were confirmed as hits at 30 μM and 4 at 10 μM in juveniles. None were confirmed as hits at the lowest tested concentration, 3.333 μM. A detailed overview of all compounds tested in juveniles and adult worms with descriptions of the phenotype is presented in Table 1. Over time viability score of compounds tested on juveniles at three concentration ranges (3.333 μM, 10 μM, and 30 μM) in S6 and S7 Figs).

**Fig 6:**
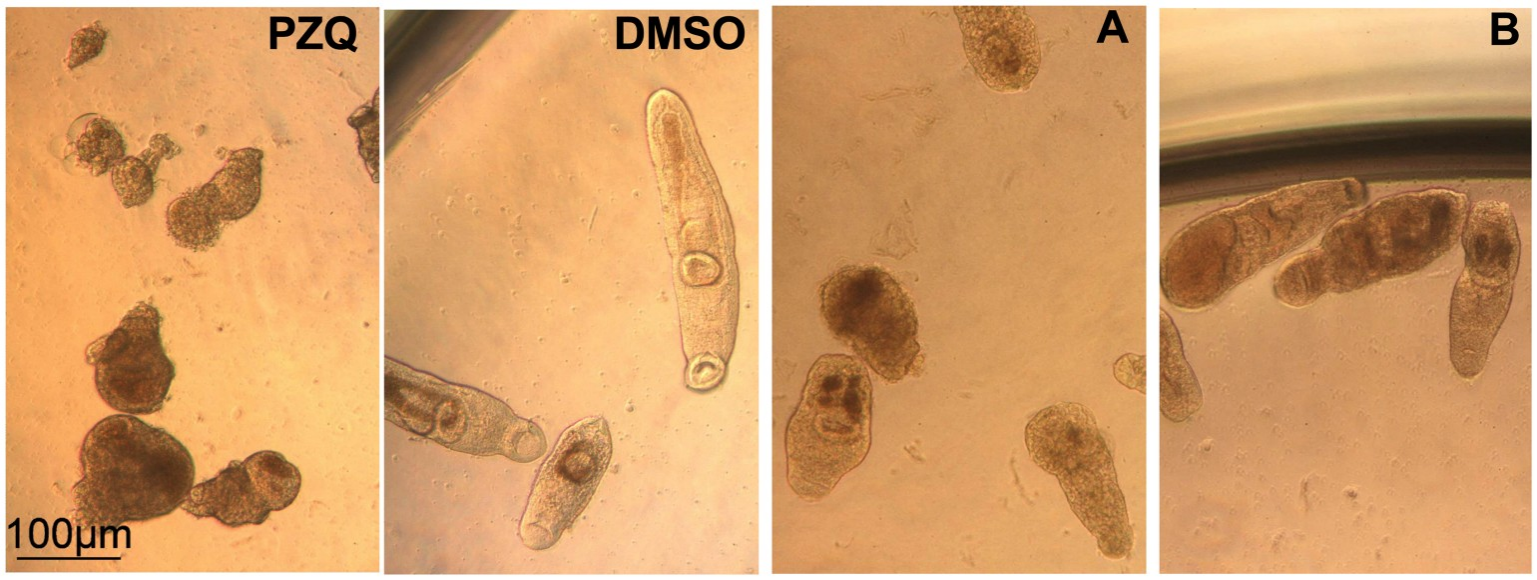
*Schistosoma mansoni* juveniles treated with PZQ: Praziquantel, DMSO: Dimethyl sulfoxide; A: Perhexiline, B: Astemizole at 24 hours (h). PZQ and compounds were tested at 30 μM and DMSO at 0.3% v/v. Images were taken at 10x magnification. DMSO: Dimethyl sulfoxide; v/v: volume by volume.

Translation from juveniles to adult worms in terms of phenotypic changes was confirmed for two compounds, perhexiline and astemizole (Table 1 and Fig 6). In juvenile worms, perhexiline induced marked changes to immature worms at 30 μM. Lower concentrations of perhexiline (3.333 μM and 10 μM) were not confirmed as hits (S6 Fig). However, at 30 μM concentration of perhexiline, liver stages displayed heavily-reduced motility and increased granularity. In some, internal structures could not be seen (Fig 6A). Viability score in two replicates at 24 hours was 0.50 and 0.75 (Table 1 and S6 Fig). The damage to juveniles was irreversible after removal of perhexiline with a further decline in viability at day 7 (168 h) (S6 Fig). Liver stages were heavily granulated, dark brown due to granulation with the presence of a bubble on the surface or disintegration of internal structures.

In the presence of 30 μM astemizole, at 24 hours, liver stages showed reduced activity, increased granulation, and darker color. Some were deformed or damaged (Fig 6B). Viability score in two replicates was 0.75 and 1.00 (Table 1 and S7 Fig). As observed in the case of perhexiline, juveniles did not recover on day 7 (168 h). Instead, their viability further declined even after removal of astemizole (S7 Fig); the majority of liver stages were densely granulated, disintegrated and dark brown, and in the ones with shape, motility was present but reduced.

**Fig 7:**
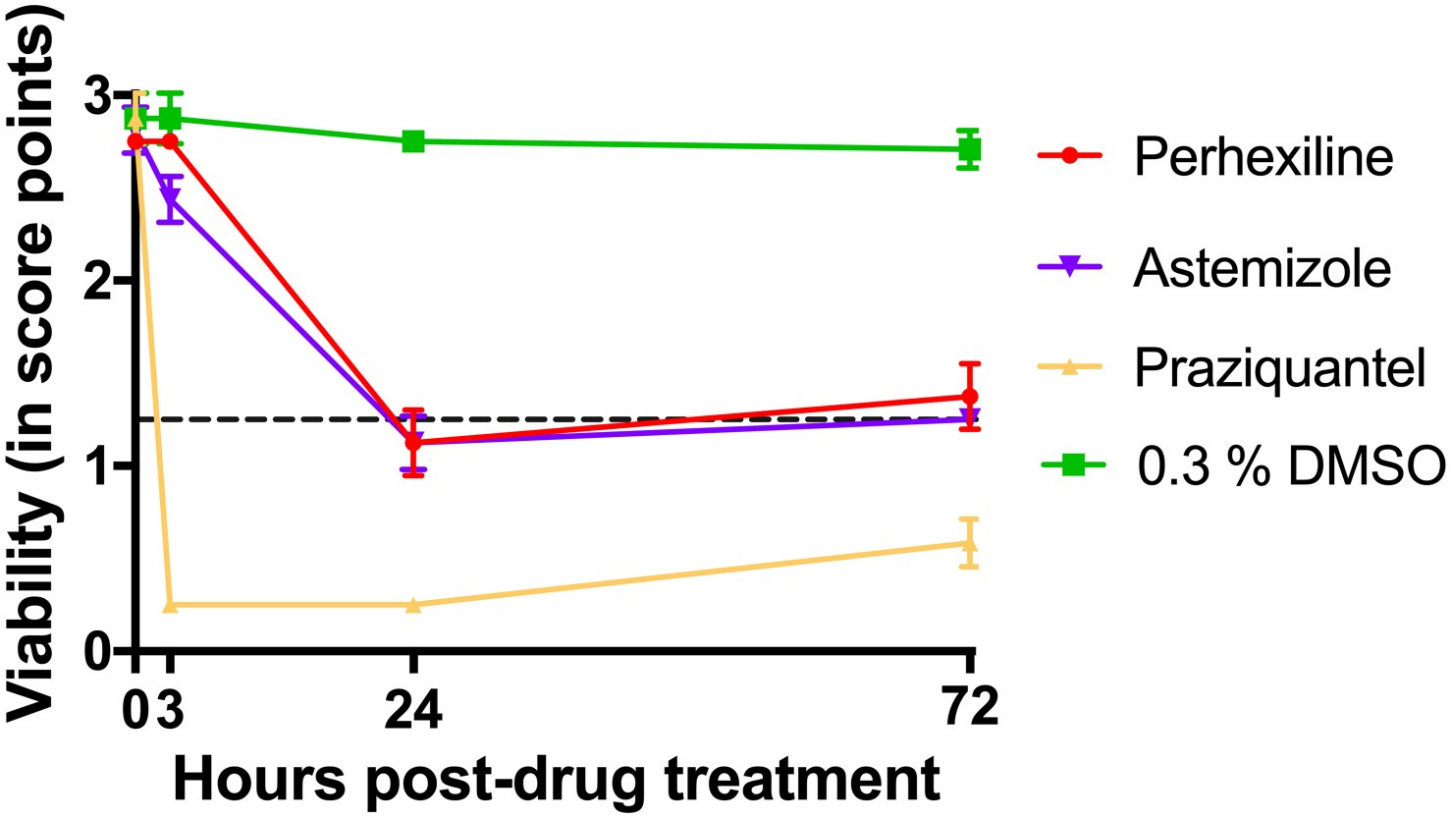
Confirmation of hits on adult *S*. *mansoni* worms. Hits were identified if an average viability score was ≤ 1.25 (dashed line). Compounds, including praziquantel (PZQ, positive control) were tested at 30 μM. DMSO (0.3% v/v) served as a negative control. Compounds and controls were removed and replaced with culture medium Hybridomed Diff 1000 supplemented with 200 U/mL penicillin, 200 μg/mL streptomycin and 20% commercial human serum (S106B-EU) 24 h post-drug treatment (p.d.t). n = 2 per compound per timepoint; n = 4 at 0 h, 3 h, and 24 h p.d.t; n = 6 PZQ and 0.3% v/v DMSO. Data shows mean ± standard deviation. DMSO: Dimethyl sulfoxide; h: hours; v/v: volume by volume.

**Fig 8:**
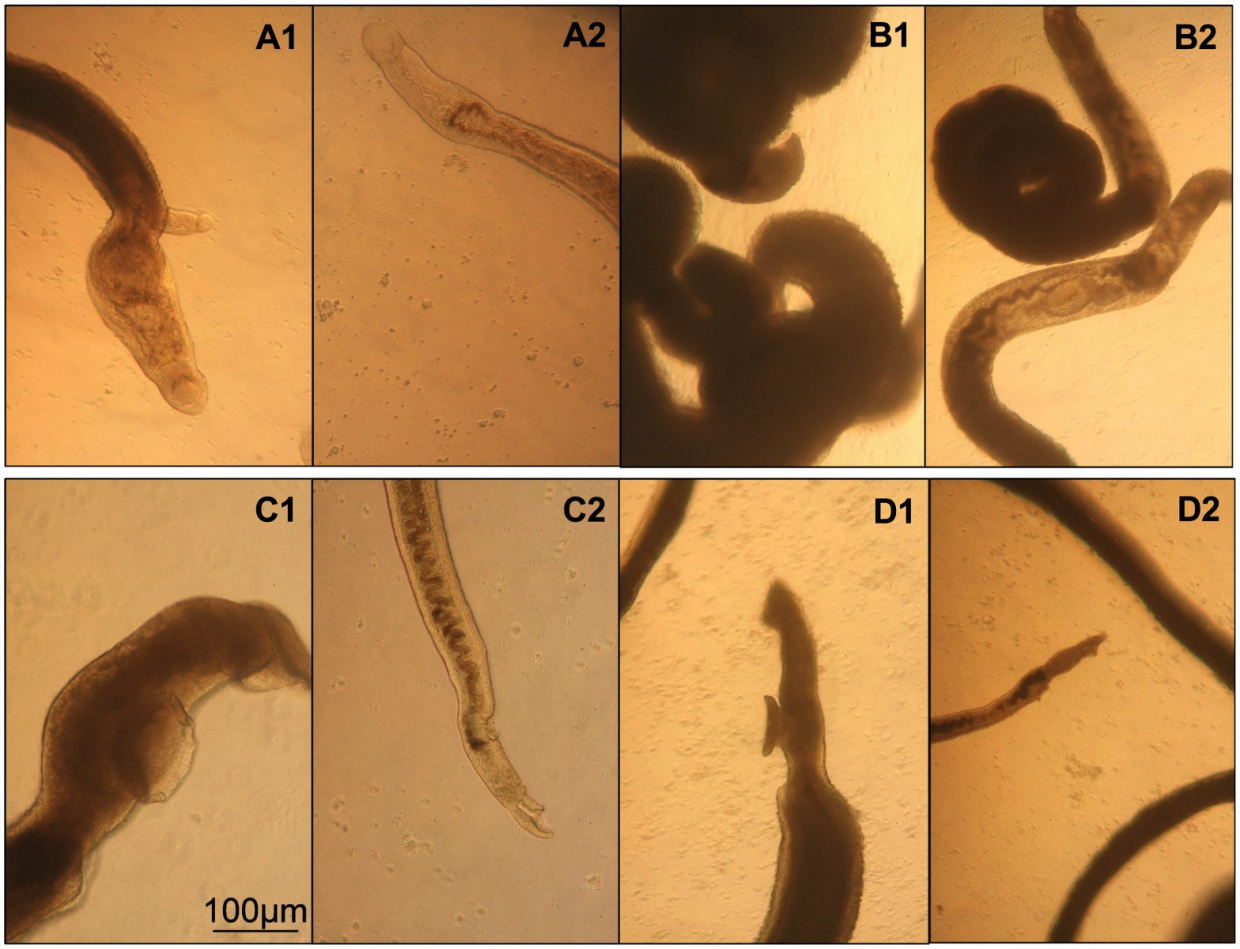
Screening of adult *Schistosoma mansoni* worms. Worms were treated at 30 μM for 24 hours with A: DMSO (0.3% v/v), A1: Paired male and female worm (5x), A2: Female worm (10x); B: Praziquantel, B1: Male worm (5x), B2: Female worm (5x); C: Perhexiline, C1: Male worm (10x), C2: Female worm (10x); D: Astemizole, D1: Male worm (5x), D2: Female worm (5x). DMSO: Dimethyl sulfoxide, x: magnification; v/v: volume by volume.

In adult worms, perhexiline showed a marked effect after 24 h (Fig 7); activity was decreased with increased granulation around oral and ventral suckers in both males (Fig 8C1) and females (Fig 8C2), especially in males. Viability score declined from 3 to 24 hours to below 1.25 (Fig 7). After the compound was washed, male and female adult worms showed a slight improvement in viability (Fig 7). Similar to perhexiline, astemizole, showed a pronounced effect on adult worms after 24-hour exposure. Worms appeared darker in color, especially males with convoluted shape and blebbing of the tegument (Fig 8D1). The area around oral and ventral suckers showed granulation in female worms (Fig 8D2). Although the average viability score for worms incubated in the presence of 30 μM astemizole was below 1.25 at 24 h, after the compound was removed, worms showed a slight recovery at 72 h (Fig 7). Although perhexiline and astemizole caused irreversible damage to juvenile worms, the two compounds did not show a detrimental effect on adult worms.

**Fig 9:**
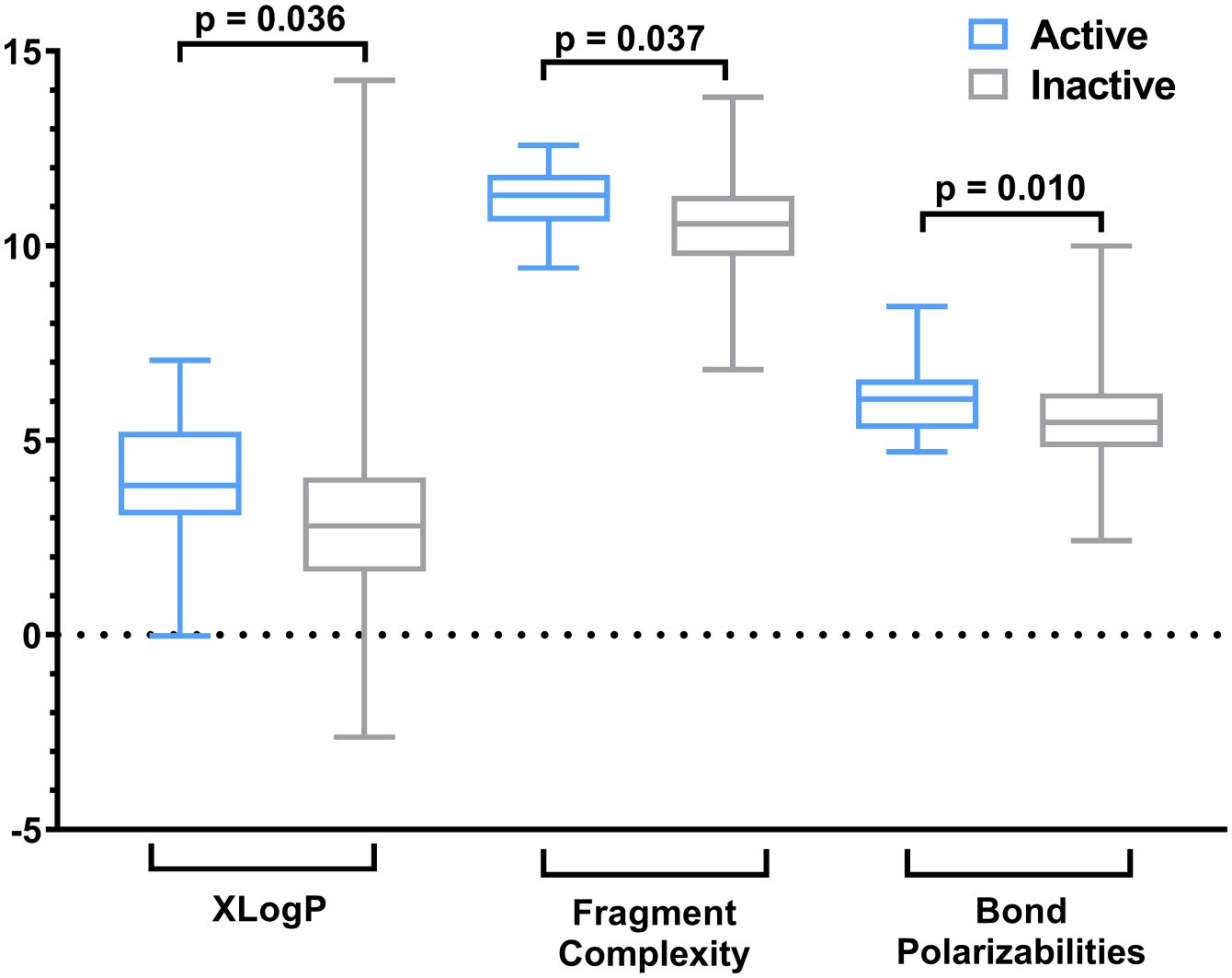
Physicochemical space of primary hits compared to inactive compounds. The active compounds showed significantly larger values of XLogP (p = 0.036), fragment complexity (p = 0.037; log2 of the actual property values are shown), and bond polarizabilities (p = 0.010; sqrt of the actual property values are shown) than the inactive compounds. P-values were calculated using Student’s t-test.

Finally, we analyzed the physicochemical space of primary hits and compared it to inactive compounds. The active compounds showed significantly larger values of XLogP, fragment complexity, and bond polarizabilities than the inactive compounds (Fig 9). This means that the active molecules are more lipophilic, structurally more complex and polarizable. Thus, these characteristics may contribute to their anti-parasitic activity.

## Discussion

We have previously reported the development of an *in vitro* assay for the generation of immature schistosomula stages up to advanced liver stages [14]. To ensure assay standardization for drug screening and to increase the throughput of compounds, the assay underwent further optimization. To achieve this, we tested different batches of commercially available medium and compared them to donor serum. Our results showed that the batch of commercial human serum plays an important role in the development of schistosomula. Even though the two sera, H6916 (serum from whole blood) and H4522 (serum from male AB plasma) were prepared differently, a similar negative effect on viability and development of schistosomula was observed for which further studies are needed to understand the underlying reasons. However, it should be noted that visually H6916 and H4522 were less translucent and milky in appearance compared to donor serum and commercial serum of EU origin (S-106B-EU). Presumably, this is due to lipid levels in H6916 and H4522. More specifically, H6916 (human serum from male AB clotted whole blood) and H4522 (serum from male AB plasma, USA origin) contained at 30–175 mg/dL (product certificate). The cholesterol levels in sera H6916 and H4522 were 80–200 mg/dL and 110–210 mg/dL, respectively. On the other hand, the commercial serum of EU origin, mixed-gender (S-106B-EU), showed a comparable and positive effect on overtime viability and development to juvenile worms. Importantly, this transition from healthy donors to commercially available serum is considered a crucial step in *in vitro* assay optimization by standardizing supply of human serum for parasite growth. Moreover, shifting to the commercial source of human sera may help in standardizing the cultivation of juveniles across different laboratories, thus enabling a more straightforward comparison of the obtained results.

Another essential improvement in the assay was downscaling from 100 to 50 NTS per well. One side effect of reducing the number of schistosomula per well was a better nutritional supply, which led to bigger individual liver stages intended for screening. Secondly, downscaling to initial 50 NTS per well led to higher throughput of screened compounds.

In our assay setting, we have observed a striking difference in the development of two *S*. *mansoni* strains, NMRI and Brazilian. Compared to the Brazilian strain, the NMRI strain had a faster pace of growth to advanced liver stages which shortened the time of cultivation for drug screening. As shown by TEM and SEM, day 29-old schistosomula (NMRI) had already developed tegument with spines, as well as appropriate size and shape and well-developed ventral and oral suckers, features common in adult worms. Already Clegg, 1965 and Basch, 1981, using SEM compared between *in vitro* cultured and *in vivo* schistosomula regarding surface features. The study showed no apparent differences in tegumental folding, morphogenesis, spines, sensory structures, or other components. Still, the study revealed that the size of the parasite in culture is 20–40% smaller than its *in vivo* counterpart [16,17]. Similarly, in our *in vitro* culture, the schistosomula showed no apparent changes in the surface features but showed a marked reduction in the size of 40% already described by these authors for the *in vitro* cultured parasites.

On the other hand, the Brazilian strain showed a slower development with the majority of worms being in lung stage at day 21, i.e., time of primary screening with the NMRI strain. Variation in the development of *S*. *mansoni* strains is not surprising. Earlier studies with infected mice showed a difference among *S*. *mansoni* strains concerning the prepatent period, egg distribution and egg numbers in tissues and feces [21,22]. The finding from our study on the pace of development shows that the protocol for *in vitro* cultivation needs to be adapted based on growth requirements for a particular *S*. *mansoni* strain before the screening of compounds. Importantly, choosing a strain with faster *in vitro* growth may be cost-effective as it decreases waiting time, labor, and volume of culture medium used prior to compound testing.

Over the years, various *in vitro* platforms were developed in an attempt to identify potential candidates for hit-to-lead programs. Such assays have employed a variety of strategies; measuring the metabolic activity and parasite motility by isothermal microcalorimetry of newly transformed schistosomula to *ex vivo* harvested 49-day old adult worms [23], measurement of parasite motility of *ex vivo* harvested adult worms by xCELLigence [24,25], an impedance-based microfluidic assay using NTS [26], employing fluorescence/luminescence-based markers in NTS [27–29], a colorimetric assay based on the tetrazolium salt XTT [30], lactate-based drug sensitivity assay [31], ATP quantification in NTS [32,33] and automated label-free, high content-based, high throughput screen to using bright-field imaging [34]. However, drug screening and repurposing was mostly done on NTS and *ex vivo* harvested adult *S*. *mansoni* adult worms. In one study, three-week-old juveniles were used in high throughput-screening of anti-schistosomal drugs; however, they were recovered by portal perfusion from infected mice [35]. The advantage of our assay is that more advanced stages, i.e., juveniles (liver stages) can be cultured under *in vitro* conditions and utilized in primary screening. Therefore, in the quest for alternatives to PZQ, drug repurposing have been extensively explored as it would allow accelerating the development timelines [36–40]. With our optimized *in vitro* assay for the generation of *S*. *mansoni* juveniles, we have screened a total of 340 approved drugs from NCATS library [15]. From primary screening, 20 hits were identified, and 13 compounds were subsequently confirmed on advanced liver stages. Primary hits were subjected to confirmation on juveniles. Our findings showed that compounds did reconfirm on juveniles with moderate potency at 10 and 30 μM, although some dose-response effects were seen from 3 to 30 μM.

Overall, the optimized *in vitro* assay was able to pick up compound active on *S*. *mansoni* juvenile worms with a primary hit rate of 5.9% from the NCATS library (340 compounds).

The use of our *in vitro* screening platform against advanced liver stages showed similarities to the screening of adult worms. As shown with TEM images, the majority of organ structures are already present and developed in 29–old schistosomula. However, translation from juveniles to adult worms in terms of the potency of compounds was limited. In our drug screening protocol using *in vitro* grown juveniles and *ex vivo* harvested adult worms, only 2 compounds transposed their activity on adult worms with moderate activity, perexiline and astemizole. Perexiline is a prophylactic antianginal agent with anti-schistosomal activity on NTS, *ex vivo* harvested juvenile and adult worms. However, *in vivo*, the compound has shown varying efficacy in decreasing worm burden [41]. The overall observed difference in the hit rate between *in vitro* juveniles and *ex vivo* adult worms is an important area to explore in order to evaluate, for example, whether the predicted targets of hit compounds are expressed in the two parasite stages. On the one hand, it is known that some compounds are specific for earlier stages with less effect on adult worms. On the other hand, fully developed adult worms show to be less phenotypically vulnerable to compounds as compared to liver stages cultured *in vitro*. What is important to note, that, both for juveniles and adult worms, human serum is supplemented in the culture medium. This is important as it better mimics *in vivo* setting where the drug binds to serum proteins, and consequently, less free drug is therefore available to exert its activity. Another advantage of our drug screening platform is that compounds are removed after 24 hours to be more representative of potential drug exposures, rather than continuous exposure of NTS and adults over the course of testing.

Taken together, our findings show that the optimized assay is robust and capable of detecting subtle differences in morphological changes induced by compounds on juveniles and *ex vivo* harvested adults. Further work on identifying biomarkers from *in vitro* grown juveniles and RNA silencing will help to elucidate the mode of action of potential leads, and aid in search of alternatives to praziquantel.

## Supporting information

S1 Fig *In vitro* larval stages from skin stage to late liver stage worms. Newly transformed schistosomula were cultured in HybridoMed Diff 1000 supplemented with 200 U/mL penicillin, 200 μg/mL streptomycin and 20% human serum. Images were taken at indicated time points at 10x magnification.
(TIF)

S2 Fig Adjustment of time of primary screening. Approximately 50 or 100 NTS were cultured in a 96-well format with HybridoMed Diff 1000 supplemented with 200 U/mL penicillin, 200 μg/mL streptomycin and 20% human serum. Viability was scored during bright field microscopy at day 21, day 28, day 35, and day 42. Data represents mean ± SD for n = 235 (days 21 and 28) and n = 88 (days 35 and 42). ***p≤0.001 comparing day 35 vs day 42, ****p≤0.0001 comparing day 28 vs day 42, and day 21 vs days 28, 35, and 42 by Kruskal-Wallis non-parametric test followed by Dunn’s multiple comparison tests. NTS: Newly transformed schistosomula; SD: Standard deviation.
(TIF)

S3 Fig Representative TEM images depicting the tegument of 29–day old schistosomula. A) * Invaginations in the tegument giving it a sponge like appearance; tegumental basal layer membrane (thick black arrows); Circular muscle fibers (thin white arrows), longitudinal muscle fibers (thin black arrows); nucleus of a large subtegumental cell (thick white arrow). B) Several well developed sub-tegumental cells (thin black arrows); mitochondria (thick black arrow). C) Erupting spines (S). The scale bars present specify the degree of magnification.
(TIF)

S4 Fig Representative TEM images of 77-day old schistosomula. A) Two well developed sub-tegumental cells (thin black arrows); containing different types of granules, large nuclei and one with prominent nucleolus. The cytoplasm is filled with inclusion bodies of varying electron density. Cytoplasmic processes (thin white arrows), of the cells interdigitating between the muscle fibers and showing inclusion bodies and numerous Mitochondria (thick black arrows). B) Well-defined spines (S) erupting from the tegument. C) Some cells show abnormal mitochondria which are enlarged, edematous and have lost cristae (thick black arrows). The scale bars show the degree of magnification.
(TIF)

S5 Fig Representative SEM images of 29-day old schistosomula. SEM representative micrographs (A-E) of 29–day old *in vitro* cultured schistosomula showing well-developed suckers (ventral and oral) and tegument. The scale bars show the degree of magnification.
(TIF)

S6 Fig Confirmation of hits on juvenile *S*. *mansoni* worms (day 21). Hits were identified if an average viability score was ≤ 1.25 (dashed line). Compounds and PZQ (positive control) were tested at 3.333 μM, 10 μM, and 30 μM. DMSO (0.3% v/v) served as a negative control. Compounds and controls were removed and replaced with culture medium HybridoMed Diff 1000 supplemented with 200 U/mL penicillin, 200 μg/mL streptomycin and 20% commercial human serum (S106B-EU) 24 h post-drug treatment (p.d.t). n = 2 per compound per concentration timepoint. Data shows mean ± standard deviation. DMSO: Dimethyl sulfoxide; PZQ: Praziquantel; v/v: volume by volume.
(TIF)

S7 Fig Confirmation of hits on juvenile *S*. *mansoni* worms (day 21). Hits were identified if an average viability score was ≤ 1.25 (dashed line). Compounds and PZQ (positive control) were tested at 3.333 μM, 10 μM, and 30 μM. DMSO (0.3% v/v) served as a negative control. Compounds and controls were removed and replaced with culture medium HybridoMed Diff 1000 supplemented with 200 U/mL penicillin, 200 μg/mL streptomycin and 20% commercial human serum (S106B-EU) 24 h post-drug treatment (p.d.t). n = 2 per compound per concentration timepoint. Data shows mean ± standard deviation. DMSO: Dimethyl sulfoxide; PZQ: Praziquantel.
(TIF)

S8 Fig *Schistosoma mansoni* juveniles treated with primary hits. A: Thioproperazine; B: Spiperone; C: Bifemelane; D: Astemizole; E: Berberine; F: Camylofin; G: Pizotyline; H: Sanguinarine, I: Praziquantel (positive control) at 24 hours (h) tested at 30 μM; J: DMSO (negative control) at 24 h tested at 0.3% v/v. Images were taken at 10x magnification. DMSO: Dimethyl sulfoxide; v/v: volume by volume.
(TIF)

S1 Table Calculation of Strictly Standardized Mean Difference (SSMD) and Z’ as quality control measures for *Schistosoma mansoni* juvenile and adult worm *in vitro* assays. (DOCX)

S2 Table List of compounds not classified as primary hits. (DOCX)
