# Supplementary figures and images for: Identification of hit compounds with anti-schistosomal activity on in vitro generated juvenile worms in cell-free medium
Source: PLoS Negl Trop Dis. 2021 May 25;15(5):e0009432. doi: 10.1371/journal.pntd.0009432 (PMC8191877; doi:10.1371/journal.pntd.0009432)

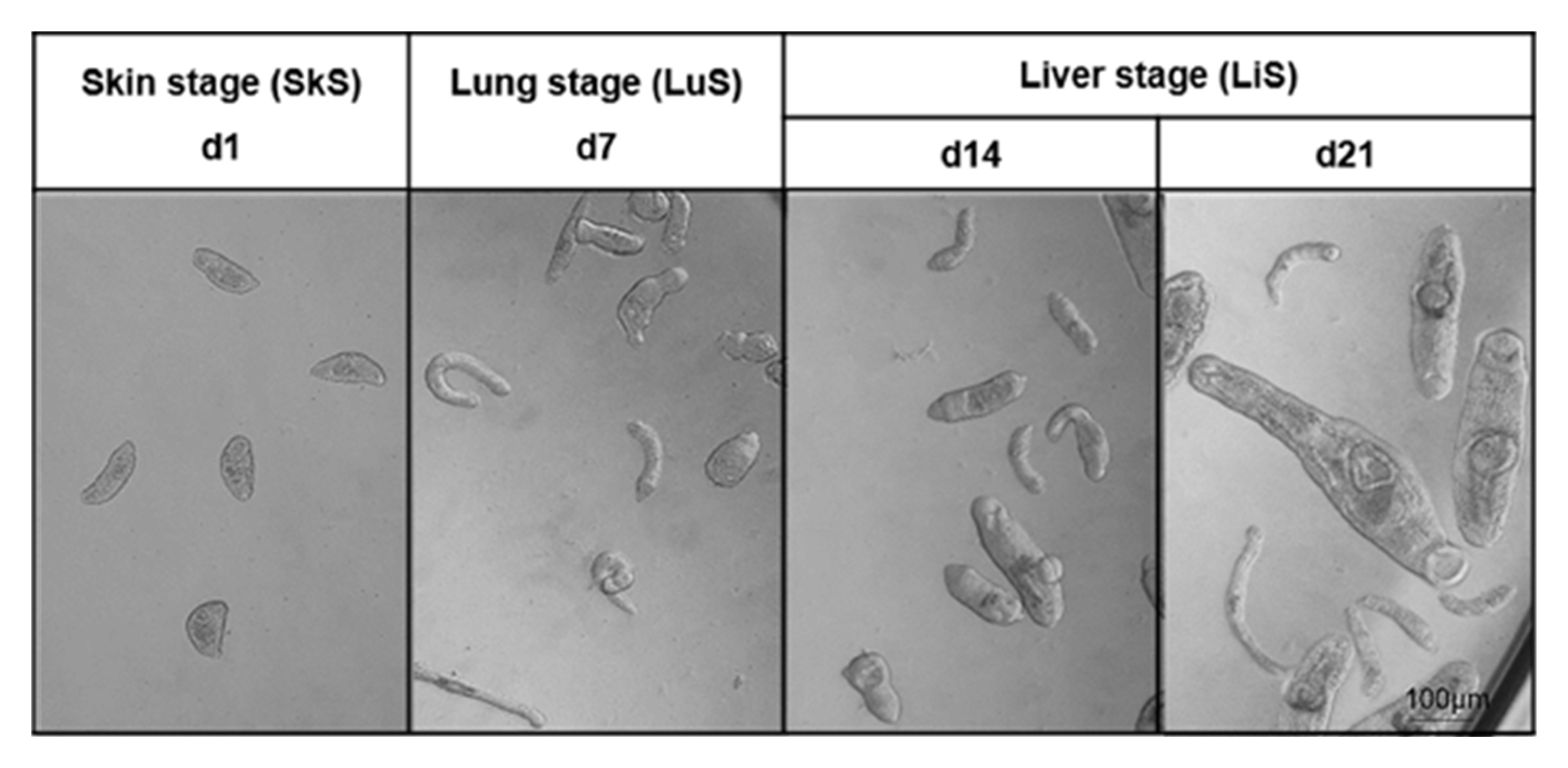

Supplement: S1 Fig — Newly transformed schistosomula were cultured in HybridoMed Diff 1000 supplemented with 200 U/mL penicillin, 200 μg/mL streptomycin and 20% human serum. Images were taken at indicated time points at 10x magnification. (TIF) [file pntd.0009432.s001.tif]

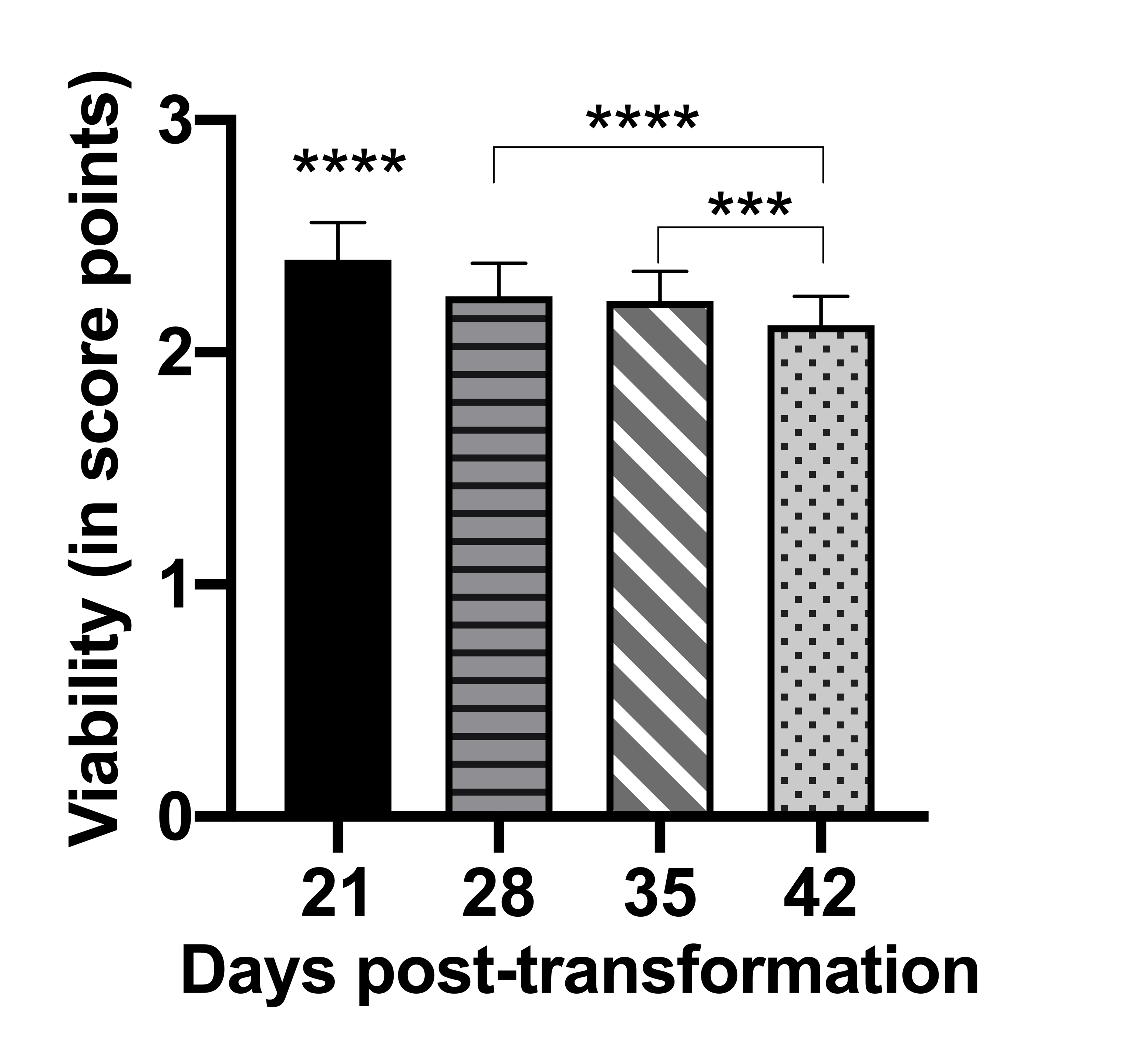

Supplement: S2 Fig — Approximately 50 or 100 NTS were cultured in a 96-well format with HybridoMed Diff 1000 supplemented with 200 U/mL penicillin, 200 μg/mL streptomycin and 20% human serum. Viability was scored during bright field microscopy at day 21, day 28, day 35, and day 42. Data represents mean ± SD for n = 235 (days 21 and 28) and n = 88 (days 35 and 42). ***p≤0.001 comparing day 35 vs day 42, ****p≤0.0001 comparing day 28 vs day 42, and day 21 vs days 28, 35, and 42 by Kruskal-Wallis non-parametric test followed by Dunn’s multiple comparison tests. NTS: Newly transformed schistosomula; SD: Standard deviation. (TIF) [file pntd.0009432.s002.tif]

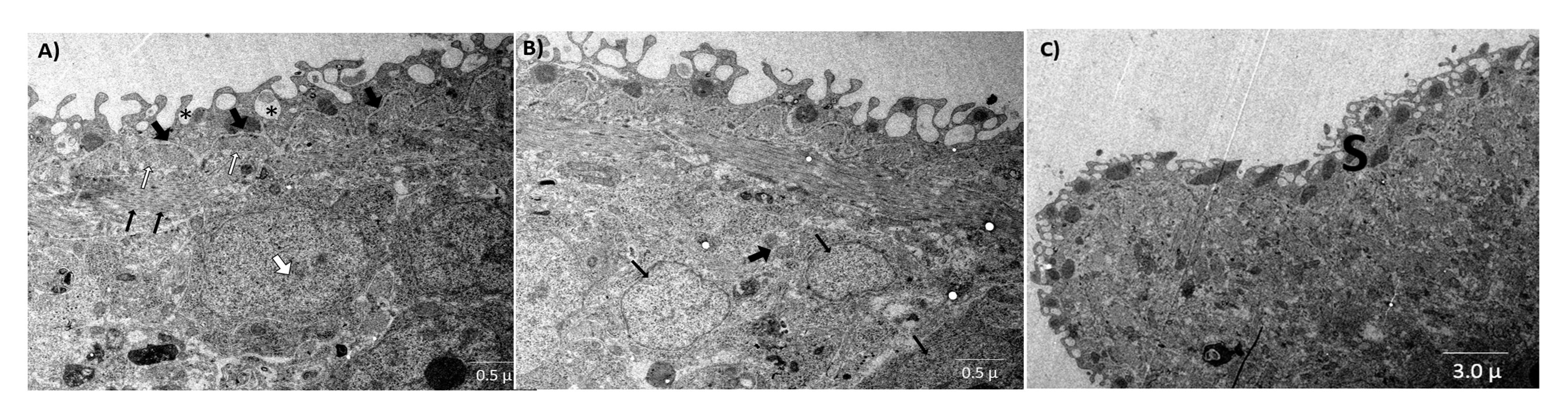

Supplement: S3 Fig — A) * Invaginations in the tegument giving it a sponge like appearance; tegumental basal layer membrane (thick black arrows); Circular muscle fibers (thin white arrows), longitudinal muscle fibers (thin black arrows); nucleus of a large subtegumental cell (thick white arrow). B) Several well developed sub-tegumental cells (thin black arrows); mitochondria (thick black arrow). C) Erupting spines (S). The scale bars present specify the degree of magnification. (TIF) [file pntd.0009432.s003.tif]

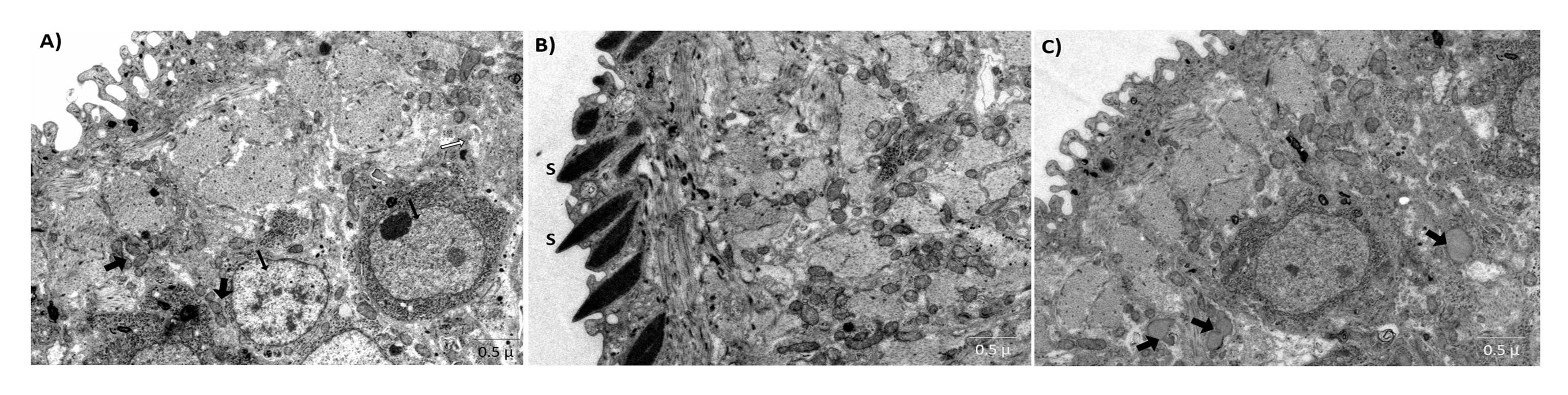

Supplement: S4 Fig — A) Two well developed sub-tegumental cells (thin black arrows); containing different types of granules, large nuclei and one with prominent nucleolus. The cytoplasm is filled with inclusion bodies of varying electron density. Cytoplasmic processes (thin white arrows), of the cells interdigitating between the muscle fibers and showing inclusion bodies and numerous Mitochondria (thick black arrows). B) Well-defined spines (S) erupting from the tegument. C) Some cells show abnormal mitochondria which are enlarged, edematous and have lost cristae (thick black arrows). The scale bars show the degree of magnification. (TIF) [file pntd.0009432.s004.tif]

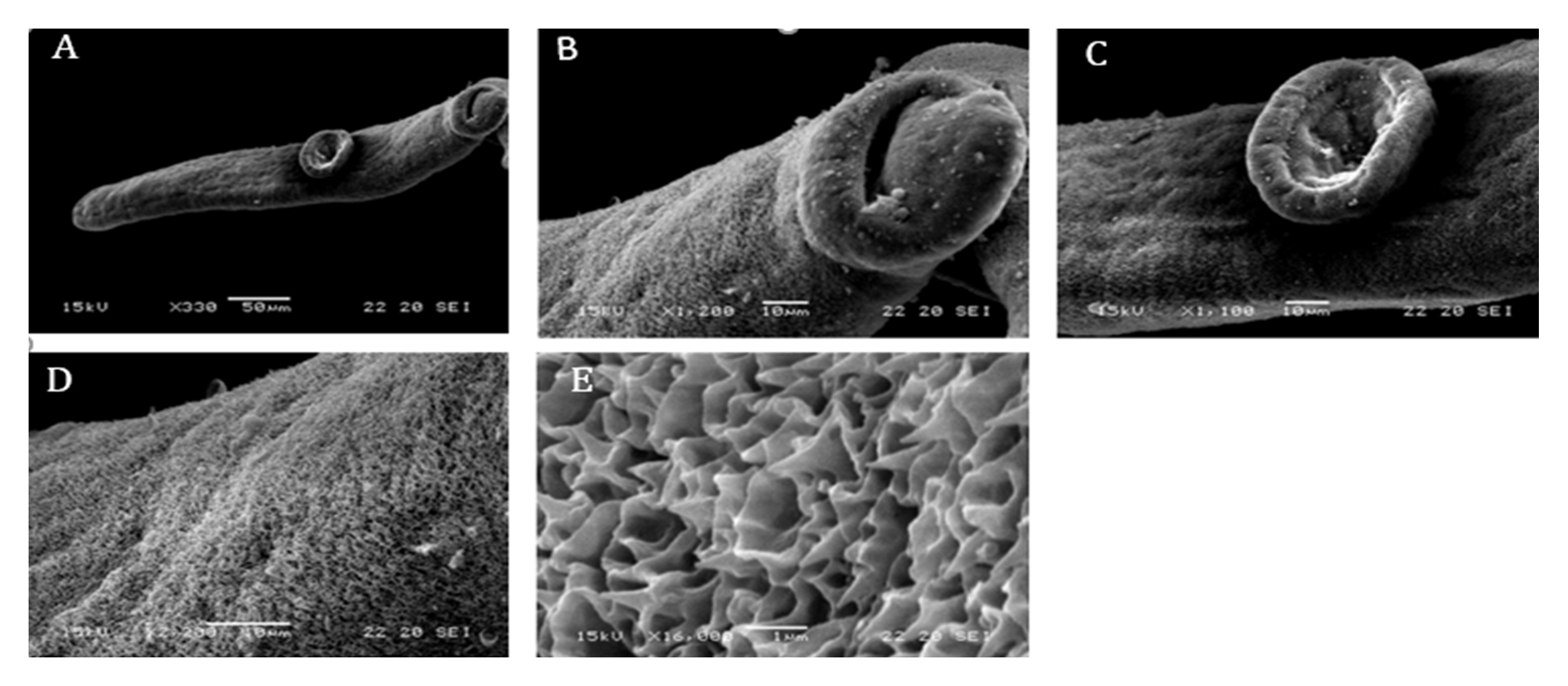

Supplement: S5 Fig — SEM representative micrographs (A-E) of 29–day old in vitro cultured schistosomula showing well-developed suckers (ventral and oral) and tegument. The scale bars show the degree of magnification. (TIF) [file pntd.0009432.s005.tif]

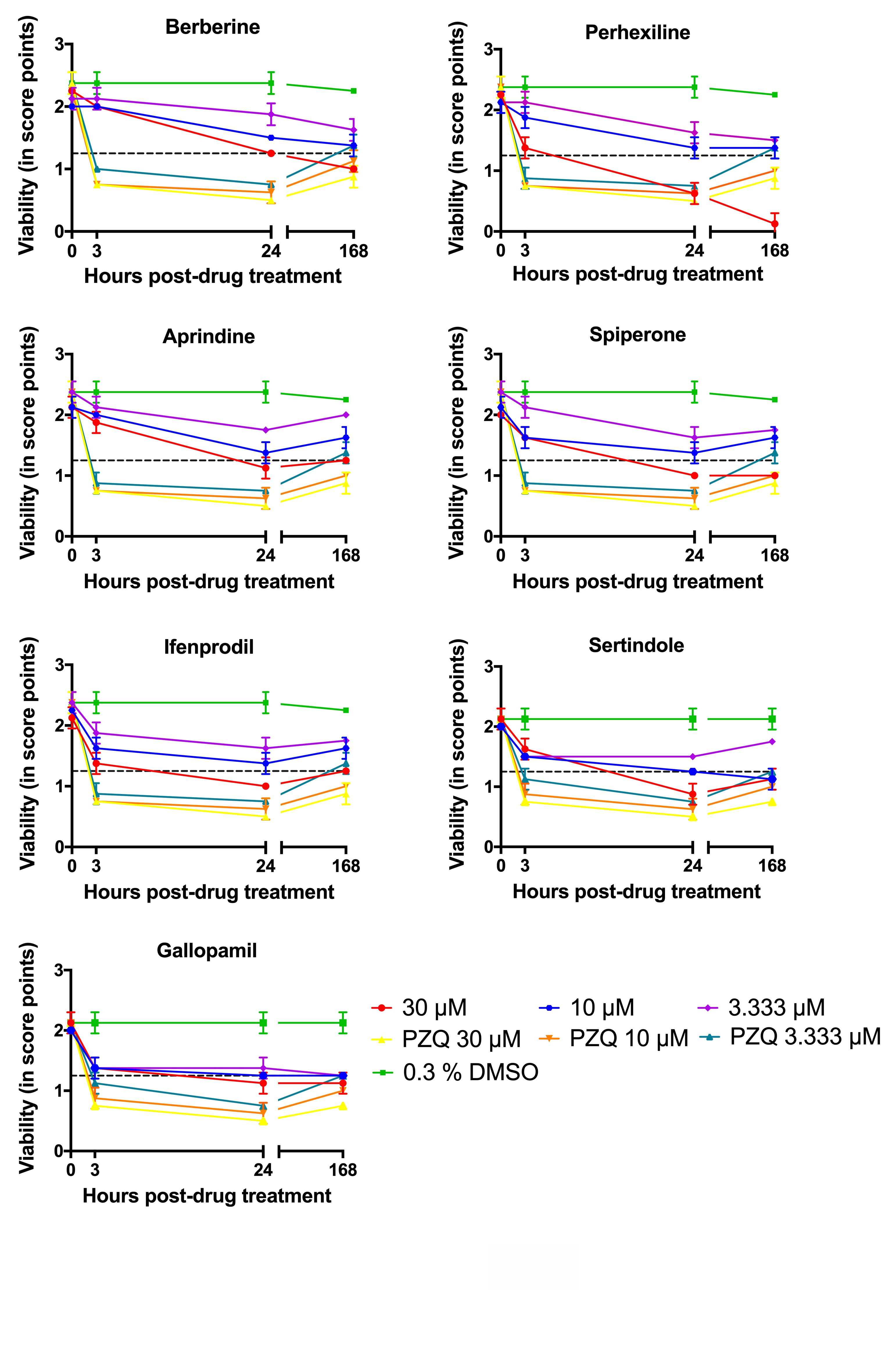

Supplement: S6 Fig — Hits were identified if an average viability score was ≤ 1.25 (dashed line). Compounds and PZQ (positive control) were tested at 3.333 μM, 10 μM, and 30 μM. DMSO (0.3% v/v) served as a negative control. Compounds and controls were removed and replaced with culture medium HybridoMed Diff 1000 supplemented with 200 U/mL penicillin, 200 μg/mL streptomycin and 20% commercial human serum (S106B-EU) 24 h post-drug treatment (p.d.t). n = 2 per compound per concentration timepoint. Data shows mean ± standard deviation. DMSO: Dimethyl sulfoxide; PZQ: Praziquantel; v/v: volume by volume. (TIF) [file pntd.0009432.s006.tif]

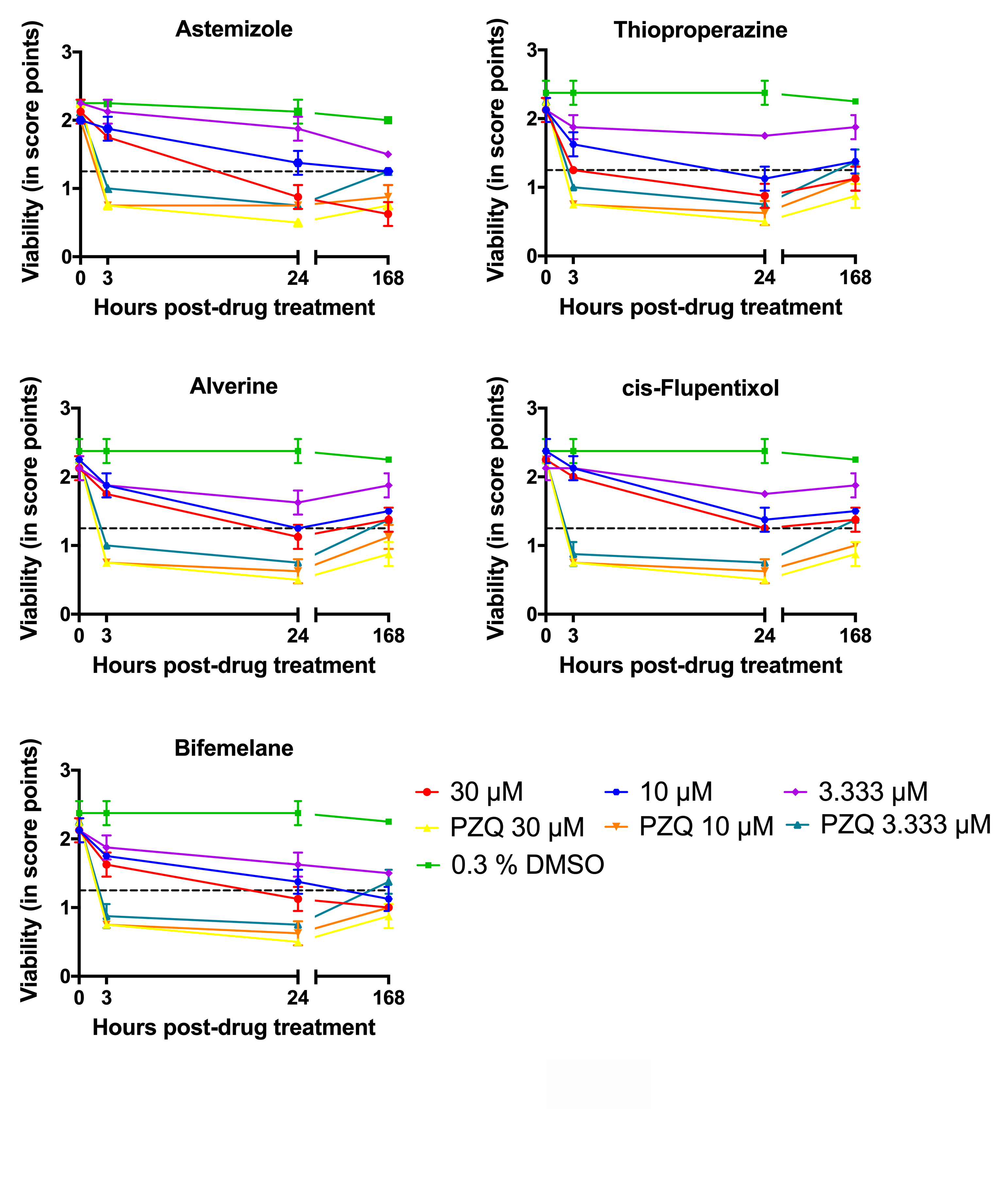

Supplement: S7 Fig — Hits were identified if an average viability score was ≤ 1.25 (dashed line). Compounds and PZQ (positive control) were tested at 3.333 μM, 10 μM, and 30 μM. DMSO (0.3% v/v) served as a negative control. Compounds and controls were removed and replaced with culture medium HybridoMed Diff 1000 supplemented with 200 U/mL penicillin, 200 μg/mL streptomycin and 20% commercial human serum (S106B-EU) 24 h post-drug treatment (p.d.t). n = 2 per compound per concentration timepoint. Data shows mean ± standard deviation. DMSO: Dimethyl sulfoxide; PZQ: Praziquantel. (TIF) [file pntd.0009432.s007.tif]

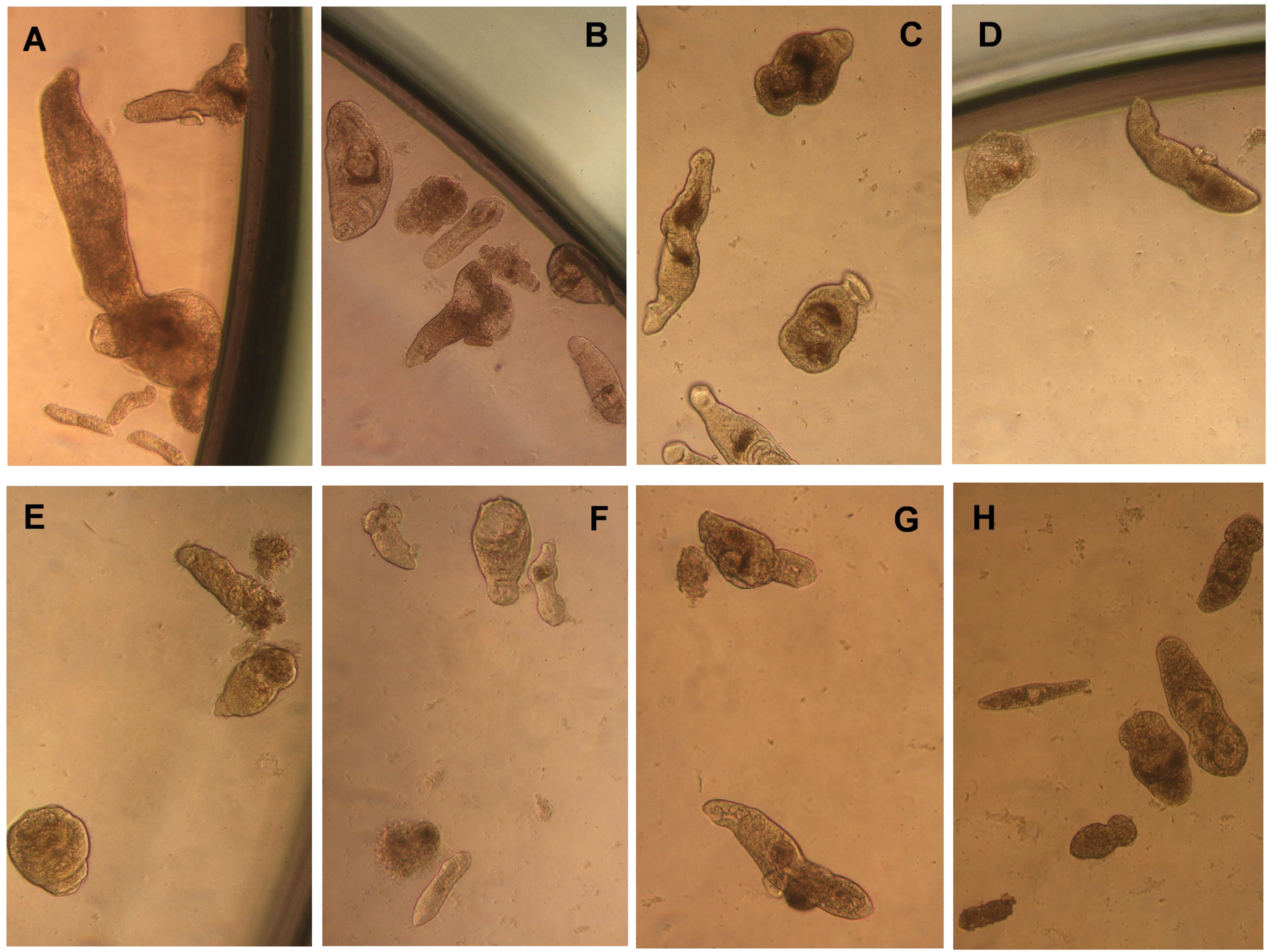

Supplement: S8 Fig — A: Thioproperazine; B: Spiperone; C: Bifemelane; D: Astemizole; E: Berberine; F: Camylofin; G: Pizotyline; H: Sanguinarine, I: Praziquantel (positive control) at 24 hours (h) tested at 30 μM; J: DMSO (negative control) at 24 h tested at 0.3% v/v. Images were taken at 10x magnification. DMSO: Dimethyl sulfoxide; v/v: volume by volume. (TIF) [file pntd.0009432.s008.tif]
