# Supplementary material for: Identification of hit compounds with anti-schistosomal activity on in vitro generated juvenile worms in cell-free medium
Source: PLoS Negl Trop Dis. 2021 May 25;15(5):e0009432. doi: 10.1371/journal.pntd.0009432 (PMC8191877; doi:10.1371/journal.pntd.0009432)
Supplement: S1 Table — (DOCX) [file pntd.0009432.s009.docx]

**S1 Table:** Calculation of Strictly Standardized Mean Difference (SSMD) and Z’ as quality control measures for *Schistosoma mansoni* juvenile and adult worm *in vitro* assays

| Hours post-drug treatment (Praziquantel/ 0.3 % DMSO) | Juvenile worms (21-42 day old); 3.333 µM PZQ | | Juvenile worms (21-42 day old); 10 PZQ | | Juvenile worms (21-42 day old); 30 µM | | Adult worm (8-9 week infection); 30 µM | |
| --- | --- | --- | --- | --- | --- | --- | --- | --- |
|  | SSMD | Z' | SSMD | Z' | SSMD | Z' | SSMD | Z' |
| 3 | 7.0  (n = 16) | 0.4  (n = 16) | 10.2  (n = 16) | 0.6  (n = 16) | 8.3  (n = 54) | 0.5  (n = 54) | 19.6  (n = 8) | 0.8  (n = 8) |
| 24 | 9.1  (n = 16) | 0.6  (n = 16) | 8.2  (n = 16) | 0.5  (n = 16) | 8.1  (n = 54) | 0.6  (n = 54) | x | 1.0  (n = 8) |
| 72 | Not determined | Not determined | Not determined | Not determined | 3.6  (n = 12) | 0.0  (n = 12) | 12.9  (n = 6) | 0.7  (n = 6) |
| Day 7 | 5.6  (n = 16) | 0.2  (n = 16) | 7.9  (n = 16) | 0.5  (n = 16) | 5  (n = 54) | 0.2  (n = 54) | 4.9  (n = 4) | 0.1  (n = 4) |

x: Calculation not possible due to the homogeneity of variance; n: Number of viability scores; DMSO: Dimethyl sulfoxide; SSMD: Strictly standardized mean difference.
