# Supplementary material for: Identification of hit compounds with anti-schistosomal activity on in vitro generated juvenile worms in cell-free medium
Source: PLoS Negl Trop Dis. 2021 May 25;15(5):e0009432. doi: 10.1371/journal.pntd.0009432 (PMC8191877; doi:10.1371/journal.pntd.0009432)
Supplement: S2 Table — (DOCX) [file pntd.0009432.s010.docx]

| **S2 Table**: List of compounds not classified as primary hits | | | | | |
| --- | --- | --- | --- | --- | --- |
| **Experiment** | **Code** | **Compound** | **Indication** | ***Schistosoma mansoni* STRAIN** | **Viability score at 24-hour post-treatment** |
| 3 | NCGC00016605-08 | Nifenazone | Pain relief | NMRI | 1.75 |
| 3 | NCGC00016697-04 | Trimetazidine dihydrochloride | Angina pectoris | NMRI | 1.50 |
| 3 | NCGC00090820-01 | 4-Acetamidobenzoic acid | Pharmaceutical preparations | NMRI | 1.75 |
| 3 | NCGC00183859-01 | Prifinium bromide | Muscarinic cholinergic receptor antagonist | NMRI | 1.50 |
| 4 | NCGC00015678-10 | Moxisylyte hydrochloride | Adrenergic receptor antagonist | Brazilian | 2.00 |
| 4 | NCGC00016089-06 | Nonivamide | Rheumatoid arthritis;muscle pain | Brazilian | 2.00 |
| 4 | NCGC00016834-07 | Fenbufen | Pain relief | Brazilian | 2.25 |
| 4 | NCGC00016878-04 | Piretanide | Hypertension;edema | Brazilian | 2.00 |
| 4 | NCGC00025195-06 | Ozagrel hydrochloride | Stroke | Brazilian | 2.25 |
| 4 | NCGC00025357-04 | Nicorandil | Angina pectoris | Brazilian | 2.25 |
| 4 | NCGC00091219-08 | N,N-Diethyl-3-methylbenzamide | Sunscreen lotion | Brazilian | 2.50 |
| 4 | NCGC00160387-04 | Clothiapine | Psychosis | Brazilian | 1.75 |
| 4 | NCGC00160444-02 | Clofenamide | Hypertension | Brazilian | 2.25 |
| 4 | NCGC00160450-03 | 4-Acetylaminophenylacetic acid | Rheumatoid arthritis | Brazilian | 2.25 |
| 4 | NCGC00160451-02 | Ibufenac | Arthritis, Rheumatoid | Brazilian | 2.00 |
| 4 | NCGC00160527-03 | Mosapride citrate | Hypertrophic gastritis (GHG); gastroesophageal reflux disease (GERD); dyspepsia; irritable bowel syndrome | Brazilian | 2.50 |
| 4 | NCGC00162385-04 | Olprinone hydrochloride | Congestive heart failure | Brazilian | 2.25 |
| 4 | NCGC00164582-02 | Feprazone | Anti-Inflammatory Agents, non-steroidal | Brazilian | 2.00 |
| 4 | NCGC00179312-03 | Cyclopenthiazide | Hypertension;congestive heart failure | Brazilian | 2.00 |
| 4 | NCGC00182995-01 | Etomidoline | Muscarinic cholinergic receptor antagonist | Brazilian | 2.25 |
| 4 | NCGC00183836-01 | Emiglitate | Alpha-glucosidase inhibitor | Brazilian | 2.00 |
| 4 | NCGC00253737-01 | Istradefylline | Parkinson's disease (PD) | Brazilian | 2.00 |
| 4 | NCGC00262941-01 | Ipenoxazone hydrochloride | Alzheimer's disease | Brazilian | 2.00 |
| 4 | NCGC00274057-01 | Phenylpropanolamine hydrochloride | Adrenergic receptor agonist | Brazilian | 2.25 |
| 4 | NCGC00344565-04 | Mizolastine | Urticaria;allergic rhinitis | Brazilian | 2.00 |
| 5 | NCGC00015099-11 | Alprenolol | Angina pectoris; | NMRI | 1.75 |
| 5 | NCGC00015810-26 | Papaverine hydrochloride | Myocardial infarction;angina pectoris;pulmonary embolism (PE);colitis;vascular spasm | NMRI | 1.75 |
| 5 | NCGC00016551-04 | Meprylcaine hydrochloride | Pain | NMRI | 2.00 |
| 5 | NCGC00018100-08 | Fasudil hydrochloride | Cerebral vasospasm | NMRI | 1.50 |
| 5 | NCGC00018117-17 | Theophylline | Asthma;bronchitis;emphysema;chronic obstructive pulmonary disease (COPD);bronchospasm | NMRI | 1.75 |
| 5 | NCGC00095188-04 | Pantoprazole | Gastroesophageal reflux disease (GERD);erosive esophagitis (EE); Zollinger-Ellison syndrome | NMRI | 2.00 |
| 5 | NCGC00159419-04 | Barbital | Sedative; seizures | NMRI | 1.75 |
| 5 | NCGC00159430-04 | Hexobarbital | Pain Disorder | NMRI | 1.75 |
| 5 | NCGC00159451-02 | Tinoridine | Anti-Inflammatory Agents, Non-Steroidal | NMRI | 1.75 |
| 5 | NCGC00160445-02 | Bromisovalum | Hypnotics and sedatives | NMRI | 1.75 |
| 5 | NCGC00160482-02 | Sulfalene | Malaria;urinary tract infections;respiratory tract infections | NMRI | 1.75 |
| 5 | NCGC00167445-02 | Eprazinone dihydrochloride | Bronchospasm | NMRI | 1.50 |
| 5 | NCGC00168759-05 | Ataluren | Duchenne muscular dystrophy (DMD);cystic fibrosis | NMRI | 1.50 |
| 5 | NCGC00181913-02 | Difeterol | Antihistamine | NMRI | 1.75 |
| 5 | NCGC00182031-01 | Etizolam | Insomnia; anxiety | NMRI | 1.75 |
| 5 | NCGC00182989-01 | Glyclopyramide | Diabetes Mellitus, Type 2 | NMRI | 1.75 |
| 5 | NCGC00183009-01 | Pranazepide | Pancreatitis | NMRI | 1.75 |
| 5 | NCGC00183011-01 | Itasetron | Memory Disorders | NMRI | 1.75 |
| 5 | NCGC00183837-01 | Monatepil | Adrenergic alpha-Antagonists;Antihypertensive Agents;Calcium Channel Blockers; | NMRI | 1.75 |
| 5 | NCGC00183871-04 | N-Acetyl sulfamethoxazole | Dihydropteroate synthase inhibitor | NMRI | 1.50 |
| 5 | NCGC00249935-01 | Phenylacetylglycine dimethylamide | Pain relief | NMRI | 1.75 |
| 6 | NCGC00013895-13 | Benzbromarone | Gout | NMRI | 2.00 |
| 6 | NCGC00015163-09 | Benserazide hydrochloride | Parkinson's Disease | NMRI | 1.50 |
| 6 | NCGC00015227-04 | Dexchlorpheniramine hydrochloride | Allergic rhinitis; urticaria; allergic conjunctivitis; allergic rhinitis; urticaria; angioedema | NMRI | 1.50 |
| 6 | NCGC00016034-05 | Tulobuterol hydrochloride | Asthma | NMRI | 1.75 |
| 6 | NCGC00016760-08 | Carbimazole | Hyperthyroidism | NMRI | 1.75 |
| 6 | NCGC00016819-05 | Gliquidone | Diabetes mellitus | NMRI | 1.75 |
| 6 | NCGC00093889-12 | Physostigmine | Hypotension; Alzheimer's disease; glaucoma; gastroparesis | NMRI | 2.00 |
| 6 | NCGC00094817-05 | Flopropione | Spasms | NMRI | 1.75 |
| 6 | NCGC00094908-01 | Mephenesin | Muscle relaxant | NMRI | 1.75 |
| 6 | NCGC00159437-03 | Clobutinol hydrochloride | Antitussive | NMRI | 2.00 |
| 6 | NCGC00160514-07 | Idebenone | Alzheimer's disease; Friedreich's ataxia; Leber hereditary optic neuropathy (LHON) | NMRI | 1.75 |
| 6 | NCGC00164492-02 | Sulfisomidine | Dihydropteroate synthase inhibitor | NMRI | 1.75 |
| 6 | NCGC00164572-14 | Rimonabant | Cannabinoid receptor antagonist | NMRI | 1.75 |
| 6 | NCGC00164601-02 | Liranaftate | Fungal infection | NMRI | 1.75 |
| 6 | NCGC00167445-02 | Eprazinone dihydrochloride | Bronchospasm | NMRI | 1.75 |
| 6 | NCGC00167454-02 | Pilsicainide | Cardiac arrythmia | NMRI | 1.75 |
| 6 | NCGC00167521-07 | Tenatoprazole | ATPase inhibitor | NMRI | 2.00 |
| 6 | NCGC00167533-04 | Vardenafil | Erectile dysfunction | NMRI | 1.75 |
| 6 | NCGC00167547-01 | Agaric acid | Anhidrotic | NMRI | 1.75 |
| 6 | NCGC00181296-02 | (+-)-7-(3,5,6-trimethyl-1,4-benzoquinon-2-yl)-7-phenylheptanoic acid | Asthma | NMRI | 1.75 |
| 6 | NCGC00182074-03 | Nicomolol | Hypolipidemic agents | NMRI | 1.50 |
| 6 | NCGC00182082-03 | Tesaglitazar | Diabetes mellitus | NMRI | 1.50 |
| 6 | NCGC00183006-01 | 1H-Indole-2-carboxamide, N-[(3R)-1-(2-fluorophenyl)-3,4,6,7-tetrahydro-4-oxopyrrolo[3,2,1-jk][1,4]benzodiazepin-3-yl]- | Pancreatitis | NMRI | 1.75 |
| 6 | NCGC00183010-01 | Pelubiprofen | Chronic pain | NMRI | 1.75 |
| 6 | NCGC00183845-01 | Pibutidine | H2-receptor antagonist | NMRI | 1.75 |
| 6 | NCGC00183852-01 | Diponium bromide | Muscarinic cholinergic receptor antagonist | NMRI | 1.75 |
| 6 | NCGC00183853-01 | Mosapramine | 5-HT2A-receptor antagonist;dopamine D2-receptor antagonist | NMRI | 1.50 |
| 6 | NCGC00183860-01 | Mefruside | Na symport inhibitor | NMRI | 1.75 |
| 6 | NCGC00242597-01 | Saxagliptin | Diabetes mellitus | NMRI | 1.75 |
| 6 | NCGC00253587-01 | Sitaxentan | Pulmonary arterial hypertension | NMRI | 1.75 |
| 6 | NCGC00254094-01 | Isoniazid | Tuberculosis | NMRI | 1.75 |
| 6 | NCGC00263583-01 | Temocapril hydrochloride | Hypertension;congestive heart failure; diabetic nephropathy; coronary artery disease (CAD) | NMRI | 1.75 |
| 7 | NCGC00015009-16 | 4-Aminopyridine | Episodic ataxias | NMRI | 1.75 |
| 7 | NCGC00015656-12 | Mianserin | Depression | NMRI | 1.75 |
| 7 | NCGC00015661-12 | Molsidomine | Coronary artery disease (CAD) | NMRI | 1.75 |
| 7 | NCGC00015760-12 | Octopamine | Trace amine associated receptor agonist | NMRI | 1.75 |
| 7 | NCGC00016660-06 | Felbinac | Rheumatoid arthritis;muscle pain | NMRI | 2.00 |
| 7 | NCGC00016951-07 | Clebopride maleate | Nausea | NMRI | 1.50 |
| 7 | NCGC00017326-05 | (1S,9R)-beta-Hydrastine | Tyrosine hydroxylase inhibitor | NMRI | 1.75 |
| 7 | NCGC00018102-12 | Flunarizine | Migraine headache; vertigo; peripheral artery disease (PAD) | NMRI | 2.00 |
| 7 | NCGC00095107-09 | Gliclazide | Diabetes mellitus | NMRI | 1.75 |
| 7 | NCGC00160424-02 | Clobenzorex hydrochloride | Obesity | NMRI | 1.75 |
| 7 | NCGC00164612-02 | Propyphenazone | Anti-inflammatory agents, non-steroidal | NMRI | 2.00 |
| 7 | NCGC00166139-03 | 3-Hydroxy-4-butyrophenetidide | Analgesic agent | NMRI | 1.75 |
| 7 | NCGC00167356-07 | Raltegravir | Human immunodeficiency virus (HIV-1) | NMRI | 1.75 |
| 7 | NCGC00167437-02 | Ramosetron hydrochloride | Nausea; vomiting; irritable bowel syndrome | NMRI | 2.00 |
| 7 | NCGC00167499-11 | Roxatidine Acetate hydrochloride | Zollinger-Ellison syndrome; erosive esophagitis (EE); gastroesophageal reflux disease (GERD); gastritis | NMRI | 2.00 |
| 7 | NCGC00167538-02 | Clorprenaline hydrochloride | Asthma | NMRI | 1.75 |
| 7 | NCGC00179630-03 | Homatropine HBr | Pupil dilation | NMRI | 2.00 |
| 7 | NCGC00181010-02 | Mabuterol hydrochloride | Asthma | NMRI | 1.75 |
| 7 | NCGC00182063-02 | Cadralazine | Hypertension | NMRI | 2.00 |
| 7 | NCGC00182987-01 | Esonarimod | Rheumatoid arthritis | NMRI | 1.75 |
| 7 | NCGC00182991-01 | Glybuzole | Type II diabetes mellitus | NMRI | 1.75 |
| 7 | NCGC00182994-01 | Anacolin | Muscarinic cholinergic receptor antagonist | NMRI | 1.75 |
| 7 | NCGC00183858-01 | Blonanserin | Schizophrenia | NMRI | 1.50 |
| 7 | NCGC00183868-02 | Fentiazac | Muscle pain; joint pain | NMRI | 2.00 |
| 7 | NCGC00249909-01 | Fenbutrazate | Obesity | NMRI | 1.75 |
| 7 | NCGC00249915-01 | Bupranolol | Hypertension; glaucoma | NMRI | 1.50 |
| 7 | NCGC00249934-01 | Tolciclate | Squalene epoxidase inhibitor | NMRI | 1.75 |
| 7 | NCGC00261971-01 | Mazaticol | Parkinson disease, secondary | NMRI | 1.75 |
| 7 | NCGC00262943-01 | Tocanfil | Biliary tract diseases | NMRI | 1.75 |
| 7 | NCGC00262961-01 | Cromoglicate lisetil | Anti-allergic agents | NMRI | 1.50 |
| 7 | NCGC00263577-01 | Loxoprofen | Pain relief | NMRI | 1.75 |
| 7 | NCGC00274083-01 | Loxiglumide | Pancreatitis | NMRI | 2.00 |
| 8 | NCGC00015243-10 | Clemizole hydrochloride | Allergic rhinitis | NMRI | 1.75 |
| 8 | NCGC00016382-07 | Oxethazaine | Local anesthetic | NMRI | 1.50 |
| 8 | NCGC00016764-04 | Benfotiamine | Lumbago | NMRI | 1.75 |
| 8 | NCGC00016889-06 | Tenoxicam | Rheumatoid arthritis;osteoarthritis;ankylosing spondylitis;tendinitis;bursitis;periarthritis; | NMRI | 1.75 |
| 8 | NCGC00018254-08 | Bifonazole | Tinea pedis; tinea cruris | NMRI | 1.75 |
| 8 | NCGC00018295-05 | Isoxsuprine hydrochloride | Raynaud's disease; arteriosclerosis; Buerger's disease | NMRI | 1.50 |
| 8 | NCGC00093362-05 | Betahistine mesylate | Meniere's disease | NMRI | 1.75 |
| 8 | NCGC00164556-02 | Fudosteine | Chest congestion | NMRI | 1.50 |
| 8 | NCGC00164578-02 | Etoricoxib | Rheumatoid arthritis; psoriatic arthritis; osteoarthritis; gout; ankylosing spondylitis | NMRI | 1.75 |
| 8 | NCGC00166397-02 | Lofepramine hydrochloride | Depression | NMRI | 1.75 |
| 8 | NCGC00167456-02 | Bisibutiamine | Asthenia | NMRI | 1.50 |
| 8 | NCGC00167519-02 | Ramatroban | Coronary artery disease (CAD); asthma | NMRI | 1.50 |
| 8 | NCGC00167524-02 | Amorolfine | Onychomycosis | NMRI | 1.75 |
| 8 | NCGC00167980-02 | Pranoprofen | Pain relief | NMRI | 1.75 |
| 8 | NCGC00178069-05 | Cloperastine hydrochloride | Cough suppressant | NMRI | 1.50 |
| 8 | NCGC00181294-01 | Thiamine disulfide | Vitamins | NMRI | 1.75 |
| 8 | NCGC00181755-01 | Delapril hydrochloride | Hypertension | NMRI | 1.75 |
| 8 | NCGC00181760-01 | Lobenzarit sodium | Rheumatoid arthritis | NMRI | 1.75 |
| 8 | NCGC00181914-01 | Alloclamide | Cough | NMRI | 1.75 |
| 8 | NCGC00182060-03 | l-Oxyfedrine hydrochloride | Angina pectoris | NMRI | 1.75 |
| 8 | NCGC00182548-02 | Flutropium bromide | Muscarinic cholinergic receptor antagonist | NMRI | 1.75 |
| 8 | NCGC00183014-01 | Sulfamethomidine | Antibacterial agent | NMRI | 1.75 |
| 8 | NCGC00183026-01 | Dicethiamine hydrochloride | Vitamin B | NMRI | 2.00 |
| 8 | NCGC00185747-02 | Niceritrol | Hyperlipidemia | NMRI | 1.75 |
| 8 | NCGC00249906-01 | Nicoboxil | Pain relief | NMRI | 1.50 |
| 8 | NCGC00262939-01 | Cycotiamine | Neurogenic bladder | NMRI | 1.50 |
| 8 | NCGC00263530-01 | Lafutidine | Duodenal ulcer disease;peptic ulcer disease (PUD) | NMRI | 1.50 |
| 8 | NCGC00263585-01 | Zaltoprofen | Fever; pain relief | NMRI | 2.00 |
| 8 | NCGC00319022-01 | Octotiamine | Vitamin B deficiency | NMRI | 1.75 |
| 8 | NCGC00344533-01 | Nalpha-Acetyl-L-glutamine | Peptic ulcer disease (PUD) | NMRI | 1.75 |
| 8 | NCGC00344562-01 | Celiprolol hydrochloride | Ehlers-Danlos syndrome (EDS) | NMRI | 1.75 |
| 9a | NCGC00015542-07 | Ibudilast | Asthma; stroke | NMRI | 2.00 |
| 9a | NCGC00015623-10 | Proglumide | CCK receptor antagonist | NMRI | 1.75 |
| 9a | NCGC00015718-12 | Naftopidil | Benign prostatic hyperplasia (BPH) | NMRI | 2.00 |
| 9a | NCGC00015836-12 | Pirenzepine | Peptic ulcer disease (PUD) | NMRI | 1.75 |
| 9a | NCGC00016064-13 | Terfenadine | Histamine receptor antagonist | NMRI | 1.75 |
| 9a | NCGC00016257-04 | 4-Dimethylaminoantipyrine | Dermatitis herpetiformis (DH) | NMRI | 2 |
| 9a | NCGC00016572-12 | Sulfamonomethoxine | Bacterial Infections | NMRI | 1.75 |
| 9a | NCGC00016715-04 | Trapidil | coronary artery disease (CAD); | NMRI | 2.00 |
| 9a | NCGC00016850-15 | Bezafibrate | Familial combined hyperlipidemia (FCHL);"Hyperlipoproteinemia, type I";"Hyperlipoproteinemia, type III;";"Hyperlipoproteinemia, type V"; | NMRI | 1.75 |
| 9a | NCGC00018204-13 | Vinpocetine | Stroke;senile dementia | NMRI | 1.75 |
| 9a | NCGC00025158-02 | (S)-(+)-Dimethindene maleate | Allergic rhinitis | NMRI | 1.75 |
| 9a | NCGC00090731-01 | 2-Phenoxyethanol | Anesthetics; anti-infective agents, local | NMRI | 2.00 |
| 9a | NCGC00091616-03 | 2-Ethoxybenzamide | Headache; fever; common cold | NMRI | 2.00 |
| 9a | NCGC00092377-04 | Apafant | Platelet activating factor receptor antagonist | NMRI | 1.75 |
| 9a | NCGC00164588-01 | Malotilate | Hepatic cirrhosis | NMRI | 1.75 |
| 9a | NCGC00164637-02 | Nifekalant hydrochloride | Ventricular arrhythmias; ventricular tachycardia (VT) | NMRI | 1.75 |
| 9a | NCGC00167529-02 | Itopride hydrochloride | Dyspepsia; gastroparesis; heartburn; nausea; vomiting; anorexia | NMRI | 1.75 |
| 9a | NCGC00179658-04 | Acenocoumarol | Deep vein thrombosis (DVT) | NMRI | 1.75 |
| 9a | NCGC00181909-01 | Sulcaine | Anesthetics, local | NMRI | 1.75 |
| 9a | NCGC00182034-01 | Teprenone | Cosmetic | NMRI | 2.00 |
| 9a | NCGC00182988-01 | Acreozast | Asthma | NMRI | 1.75 |
| 9a | NCGC00182990-01 | Tandospirone | Generalized anxiety disorder (GAD); dysthymic disorder | NMRI | 2.00 |
| 9a | NCGC00183005-01 | Zanapezil fumerate | Alzheimer's disease | NMRI | 1.75 |
| 9a | NCGC00183008-01 | Israpafant | Asthma | NMRI | 2.00 |
| 9a | NCGC00183020-01 | Minopafant | Asthma | NMRI | 1.75 |
| 9a | NCGC00247711-01 | Amobarbital | GABAA-receptor (picrotoxin binding site) agonist | NMRI | 2.00 |
| 9a | NCGC00249908-01 | Mefenorex | Obesity | NMRI | 1.75 |
| 9a | NCGC00249916-01 | Dimorpholamine | Respiratory insufficiency | NMRI | 1.75 |
| 9a | NCGC00249917-01 | Cloxazolam | GABAA-receptor (benzodiazepine binding site) agonist | NMRI | 2.00 |
| 9a | NCGC00249931-01 | Docarpamine | Heart failure | NMRI | 1.75 |
| 9a | NCGC00253608-01 | Chlorexolone | Hypertension | NMRI | 1.75 |
| 9a | NCGC00262929-03 | BIBR-1048 | Stroke; systemic embolism; pulmonary embolism (PE); deep vein thrombosis (DVT) | NMRI | 2.00 |
| 9a | NCGC00263447-01 | alpha-Santonin | Ascaris lumbricoides | NMRI | 1.75 |
| 9b | NCGC00015725-15 | Nimesulide | Osteoarthritis; menstrual pain | NMRI | 1.75 |
| 9b | NCGC00016353-08 | Butyl 4-aminobenzoate | Sodium channel blocker | NMRI | 2.00 |
| 9b | NCGC00018121-07 | Proxyphylline | Asthma | NMRI | 2.00 |
| 9b | NCGC00095049-04 | Exalamide | Membrane permeability inhibitor | NMRI | 2.00 |
| 9b | NCGC00163240-01 | Neostigmine bromide | Myasthenia gravis | NMRI | 1.75 |
| 9b | NCGC00164543-02 | Lomerizine dihydrochloride | migraine headache | NMRI | 2.00 |
| 9b | NCGC00164584-03 | Cetraxate hydrochloride | Anti-ulcer agents | NMRI | 1.75 |
| 9b | NCGC00167489-03 | Sarpogrelate hydrochloride | Diabetes mellitus; angina pectoris; Raynaud's disease; atherosclerosis; Buerger's disease | NMRI | 2.00 |
| 9b | NCGC00167574-03 | Xaliproden hydrochloride | Serotonin receptor agonist | NMRI | 2.00 |
| 9b | NCGC00167737-02 | Lazabemide hydrochloride | Monoamine oxidase inhibitor | NMRI | 1.75 |
| 9b | NCGC00181000-01 | Suplatast tosylate | Kimura's disease | NMRI | 1.75 |
| 9b | NCGC00181340-02 | Azosemide | Edema | NMRI | 1.75 |
| 9b | NCGC00181356-02 | Oxapium iodide | Muscarinic cholinergic receptor antagonist | NMRI | 1.75 |
| 9b | NCGC00182704-03 | Luliconazole | Tinea pedis; tinea cruris; tinea corporis | NMRI | 1.75 |
| 9b | NCGC00183012-01 | Penflutizide | Na symport inhibitor | NMRI | 2.25 |
| 9b | NCGC00186628-02 | Butylscopolammonium bromide | Abdominal pain | NMRI | 1.75 |
| 9b | NCGC00256497-01 | 4-Chlorophenol | Dental pulp diseases | NMRI | 1.75 |
| 9b | NCGC00262907-01 | Bilastine | Conjunctivitis | NMRI | 2.00 |
| 10a | NCGC00015116-13 | Aniracetam | Glutamate receptor agonist | NMRI | 1.75 |
| 10a | NCGC00015649-08 | Moxonidine | Hypertension | NMRI | 2.00 |
| 10a | NCGC00015774-06 | Oxatomide | Allergic rhinitis | NMRI | 2.00 |
| 10a | NCGC00016345-08 | 4-Methylumbelliferone | Monoamine oxidase inhibitor | NMRI | 2.00 |
| 10a | NCGC00016397-04 | Benzydamine hydrochloride | Mouth inflammation; throat inflammation | NMRI | 1.75 |
| 10a | NCGC00016743-08 | Piromidic acid | Malaria, Falciparum | NMRI | 1.75 |
| 10a | NCGC00016957-05 | Aceclofenac | Rheumatoid arthritis; ankylosing spondylitis; osteoarthritis; periarthritis; lumbago; ischiadynia | NMRI | 1.75 |
| 10a | NCGC00017259-07 | Nicergoline | Raynaud's disease; migraine headache; atherosclerosis; thrombosis | NMRI | 1.75 |
| 10a | NCGC00018187-09 | Cinnarizine | Meniere's disease; nausea; vomiting; Cogan's syndrome | NMRI | 1.75 |
| 10a | NCGC00018291-03 | Procaterol | Asthma | NMRI | 2.00 |
| 10a | NCGC00024504-09 | Kainic acid | Kainate receptor agonist | NMRI | 1.50 |
| 10a | NCGC00159473-05 | (-)-Fenfluramine hydrochloride | Serotonin receptor agonist | NMRI | 1.75 |
| 10a | NCGC00159481-03 | Sofalcone | Peptic ulcer disease (PUD) | NMRI | 1.75 |
| 10a | NCGC00160484-02 | Etafenone | Calcium channel blocker | NMRI | 1.75 |
| 10a | NCGC00161414-03 | Tropisetron | Nausea; vomiting | NMRI | 1.75 |
| 10a | NCGC00164603-04 | Ebastine | Allergic rhinitis;urticaria | NMRI | 2.00 |
| 10a | NCGC00167447-02 | Prosultiamine | Vitamins | NMRI | 2.00 |
| 10a | NCGC00167453-02 | Clinofibrate | Hypertriglyceridemia | NMRI | 1.75 |
| 10a | NCGC00167455-02 | Zofenopril | Hypertension | NMRI | 2.00 |
| 10a | NCGC00167509-02 | Pipethanate ethylbromide | Gastritis | NMRI | 1.75 |
| 10a | NCGC00167577-01 | Sivelestat sodium tetrahydrate | Acute lung injury | NMRI | 1.75 |
| 10a | NCGC00181103-02 | Propiverine hydrochloride | Urinary incontinence | NMRI | 1.75 |
| 10a | NCGC00182037-01 | Alacepril | Hypertension | NMRI | 1.75 |
| 10a | NCGC00182709-03 | Landiolol hydrochloride | Cardiac arrythmia | NMRI | 2.00 |
| 10a | NCGC00182982-01 | Dalcotidine | H2-receptor antagonist | NMRI | 1.75 |
| 10a | NCGC00183029-02 | Troxipide | Gastroesophageal reflux disease (GERD) | NMRI | 2.25 |
| 10a | NCGC00185744-02 | Methyldopate hydrochloride | Hypertension | NMRI | 1.75 |
| 10a | NCGC00188429-02 | Acitazanolast | Allergic rhinitis | NMRI | 2.00 |
| 10a | NCGC00249929-01 | Neticonazole | Sterol 14alpha-demethylase inhibitor | NMRI | 1.75 |
| 10a | NCGC00253646-01 | Agomelatine | Depression | NMRI | 1.75 |
| 10a | NCGC00253658-08 | Dapoxetine (hydrochloride) | Premature ejaculation | NMRI | 1.50 |
| 10a | NCGC00262958-01 | Bravavir | Herpes zoster | NMRI | 1.75 |
| 10b | NCGC00015821-02 | Piracetam | Senile dementia | NMRI | 2.25 |
| 10b | NCGC00016561-05 | Meticrane | Hypertension | NMRI | 2.25 |
| 10b | NCGC00016666-01 | Hydrocotarnine hydrobromide | Analgesic agent | NMRI | 2.00 |
| 10b | NCGC00016690-01 | Tribenoside | Anti-inflammatory agent; capillary stabilizing agent | NMRI | 2.00 |
| 10b | NCGC00016737-04 | Epirizole | Pain relief | NMRI | 2.00 |
| 10b | NCGC00016815-01 | Tiaprofenic acid | Rheumatoid arthritis | NMRI | 1.75 |
| 10b | NCGC00016868-12 | Acemetacin | Osteoarthritis | NMRI | 2.00 |
| 10b | NCGC00016909-01 | Glafenine hydrochloride | DeltaF508-CFTR correctors | NMRI | 2.25 |
| 10b | NCGC00017010-01 | Methylatropine nitrate | Pyloric stenosis | NMRI | 2.00 |
| 10b | NCGC00018241-07 | Betamipron | Renal toxicity | NMRI | 1.75 |
| 10b | NCGC00163160-01 | Talipexole | Genitial herpes | NMRI | 2.25 |
| 10b | NCGC00164605-09 | Ampiroxicam | Arthritis, rheumatoid | NMRI | 2.00 |
| 10b | NCGC00167448-02 | Trepibutone | Irritable bowel syndrome | NMRI | 1.75 |
| 10b | NCGC00167522-02 | Hexoprenaline sulfate | Asthma | NMRI | 1.75 |
| 10b | NCGC00178060-03 | Tolperisone hydrochloride | Muscle relaxant; multiple sclerosis; encephalomyelitis; ankylosing spondylitis; atherosclerosis; Raynaud's disease | NMRI | 2.00 |
| 10b | NCGC00178319-06 | Thiamphenicol | Bacterial Infections | NMRI | 2.00 |
| 10b | NCGC00178520-03 | Bromhexine hydrochloride | Chest congestion | NMRI | 1.75 |
| 10b | NCGC00181009-02 | Timepidium bromide | Muscarinic cholinergic receptor antagonist | NMRI | 1.50 |
| 10b | NCGC00181765-01 | Pranlukast | Bronchospasm; asthma; bronchospasm; asthma | NMRI | 1.75 |
| 10b | NCGC00181911-01 | Bucolome | Pain relief | NMRI | 2.00 |
| 10b | NCGC00182996-01 | Pamicogrel | Cyclooxygenase-1 (COX-1) inhibitor;cyclooxygenase-2 (COX-2) inhibitor; | NMRI | 2.00 |
| 10b | NCGC00182997-01 | Melinamide | Hyperlipoproteinemia, type III | NMRI | 1.75 |
| 10b | NCGC00183003-01 | Depolipon | Anesthetic | NMRI | 1.75 |
| 10b | NCGC00183024-01 | Proglumetacin | Pain relief | NMRI | 1.75 |
| 10b | NCGC00183104-02 | Nizofenone | Stroke | NMRI | 2.00 |
| 10b | NCGC00183830-01 | Lemildipine | Calcium Channel Blockers | NMRI | 2.00 |
| 10b | NCGC00249907-01 | Sulthiame | Episodic ataxias | NMRI | 1.75 |
| 10b | NCGC00249924-01 | Ibuprofen piconol | Pain relief | NMRI | 1.50 |
| 10b | NCGC00249927-01 | Simetride | Pain relief | NMRI | 2.00 |
| 10b | NCGC00249930-01 | Tolycaine | Anesthetics, local | NMRI | 1.75 |
| 10b | NCGC00249938-01 | Glymidine sodium | Diabetes mellitus | NMRI | 1.75 |
| 10b | NCGC00253611-01 | Isoaminile | Cough | NMRI | 1.50 |
| 10b | NCGC00253867-02 | Prucalopride | Constipation | NMRI | 1.75 |
| 10b | NCGC00274275-01 | Tripamide | Diuretic | NMRI | 2.00 |
| 11a | NCGC00015430-06 | Fenoterol hydrobromide | Adrenergic receptor agonist | NMRI | 2.25 |
| 11a | NCGC00015716-09 | Nylidrin | Raynaud's disease | NMRI | 1.50 |
| 11a | NCGC00015898-15 | Rolipram | Phosphodiesterase inhibitor | NMRI | 1.75 |
| 11a | NCGC00015966-10 | Levosulpiride | Schizophrenia; psychosis; anxiety; vertigo; dyspepsia; irritable bowel syndrome; premature ejaculation (PE); schizophrenia | NMRI | 2.00 |
| 11a | NCGC00016066-14 | Urapidil hydrochloride | Hypertension | NMRI | 2.00 |
| 11a | NCGC00016611-10 | Bufexamac | Cyclooxygenase inhibitor | NMRI | 1.75 |
| 11a | NCGC00016640-06 | Todralazine hydrochloride | Hypertension | NMRI | 2.25 |
| 11a | NCGC00017331-08 | (-)-Epicatechin | Fatty acid synthase inhibitor | NMRI | 1.75 |
| 11a | NCGC00018097-06 | Paroxypropione | Gonadotropin inhibitor | NMRI | 2.25 |
| 11a | NCGC00018271-07 | Homochlorcyclizine | Allergic rhinitis | NMRI | 1.75 |
| 11a | NCGC00092310-06 | Amisulpride | Psychosis; bipolar disorder; schizophrenia | NMRI | 1.75 |
| 11a | NCGC00160540-03 | Ethiazide | Diuretic | NMRI | 2.00 |
| 11a | NCGC00160628-03 | Rutin trihydrate | Joint pain; nasal congestion; constipation; irritability; allergic rhinitis | NMRI | 2.00 |
| 11a | NCGC00164512-02 | Guaiacol carbonate | Disinfectants | NMRI | 2.00 |
| 11a | NCGC00164595-02 | Voglibose | Diabetes mellitus; hyperglycemia |  | 1.75 |
| 11a | NCGC00165864-04 | Pipamperone dihydrochloride | Schizophrenia |  | 1.50 |
| 11a | NCGC00167471-01 | Anethole trithione | Glutathione transferase stimulant | NMRI | 2.25 |
| 11a | NCGC00167526-02 | Camostat mesylate | Pancreatitis | NMRI | 1.75 |
| 11a | NCGC00167973-02 | Eperisone hydrochloride | Amyotrophic lateral sclerosis (ALS); spasms; head injury; spinal injury | NMRI | 1.75 |
| 11a | NCGC00178213-05 | Nafronyl oxalate | Claudication | NMRI | 1.75 |
| 11a | NCGC00179663-03 | (-)-(5S,8R)-Lisuride | Parkinson's disease | NMRI | 2.25 |
| 11a | NCGC00181039-01 | Ethyl linoleate | Alcohol drinking | NMRI | 2.00 |
| 11a | NCGC00181795-02 | Lumiracoxib | Cyclooxygenase inhibitor | NMRI | 1.75 |
| 11a | NCGC00182081-04 | Zotepine | Schizophrenia | NMRI | 1.75 |
| 11a | NCGC00182983-01 | Fezatione | Antifungal agents | NMRI | 2.00 |
| 11a | NCGC00183007-01 | Risarestat | Diabetes complications | NMRI | 1.75 |
| 11a | NCGC00188943-03 | Orotic acid | Hepatitis | NMRI | 2.00 |
| 11a | NCGC00247710-02 | Dexfenfluramine hydrochloride | Serotonin receptor agonist | NMRI | 1.75 |
| 11a | NCGC00249416-01 | Oberadilol | Adrenergic beta-antagonists | NMRI | 2.00 |
| 11a | NCGC00249914-01 | Bencyclane | Peripheral artery disease (PAD) | NMRI | 2.00 |
| 11a | NCGC00253606-01 | Mexazolam | Anxiety | NMRI | 2.00 |
| 11a | NCGC00261968-01 | Bufetolol | Beta1-adrenergic receptor antagonist; beta2-adrenergic receptor antagonist; beta3-adrenergic receptor antagonist | NMRI | 1.75 |
| 11a | NCGC00261973-01 | Butoctamide semisuccinate | Insomnia | NMRI | 2.00 |
| 11a | NCGC00263541-01 | Reboxetine mesylate | Depression | NMRI | 2.25 |
| 11a | NCGC00263571-01 | Bendazac sodium | Muscle pain; joint pain | NMRI | 2.00 |
| 11b | NCGC00013289-09 | Pridinol | Muscle relaxant | NMRI | 1.75 |
| 11b | NCGC00014670-17 | Domperidone | Fescue toxicosis | NMRI | 1.75 |
| 11b | NCGC00015279-08 | Meclofenoxate hydrochloride | Senile dementia; Alzheimer's disease | NMRI | 1.75 |
| 11b | NCGC00015861-09 | Propentofylline | Stroke | NMRI | 2.00 |
| 11b | NCGC00015996-10 | Tiapride hydrochloride | Dyskinesia; abstinence from alcohol; psychosis | NMRI | 2.00 |
| 11b | NCGC00016367-08 | Succinylsulfathiazole | Dihydrofolate reductase inhibitor | NMRI | 2.50 |
| 11b | NCGC00018277-06 | Mebeverine hydrochloride | Irritable bowel syndrome | NMRI | 2.00 |
| 11b | NCGC00025297-04 | Gabexate mesilate | Pancreatitis | NMRI | 2.00 |
| 11b | NCGC00027930-07 | Moclobemide | Depression; anxiety | NMRI | 1.75 |
| 11b | NCGC00091120-13 | Griseofulvin | Ringworm; tinea pedis | NMRI | 2.00 |
| 11b | NCGC00095164-04 | Prothionamide | Tuberculosis | NMRI | 2.00 |
| 11b | NCGC00160398-05 | Nafamostat mesylate | Anticoagulent | NMRI | 2.25 |
| 11b | NCGC00162068-06 | Opipramol dihydrochloride | Generalized anxiety disorder (GAD) | NMRI | 1.75 |
| 11b | NCGC00164577-02 | Nimetazepam | GABAA-receptor (benzodiazepine binding site) agonist | NMRI | 2.00 |
| 11b | NCGC00164589-02 | Phenprobamate | Anxiety; muscle relaxant | NMRI | 1.75 |
| 11b | NCGC00178395-04 | Piperidolate hydrochloride | Spasms | NMRI | 1.75 |
| 11b | NCGC00181124-02 | Sparteine | Arrhythmias, cardiac | NMRI | 1.75 |
| 11b | NCGC00182710-01 | Flurbiprofen axetil | Cyclooxygenase-1 (COX-1) inhibitor; cyclooxygenase-2 (COX-2) inhibitor | NMRI | 2.00 |
| 11b | NCGC00182986-01 | Acetylpheneturide | Epilepsy | NMRI | 2.00 |
| 11b | NCGC00183000-01 | Lanperisone hydrochloride | Muscle relaxation | NMRI | 1.75 |
| 11b | NCGC00183833-01 | Bimosiamose | Synthetic pan-selectin antagonist | NMRI | 2.00 |
| 11b | NCGC00249911-01 | Normethadone | Pain relief | NMRI | 2.00 |
| 11b | NCGC00249926-01 | Pirmenol | Voltage-gated sodium channel (SCN1A) blocker; voltage-gated sodium channel (SCN2A) blocker; voltage-gated sodium channel (SCN3A) blocker; voltage-gated sodium channel (SCN4A) blocker; voltage-gated sodium channel (SCN5A) blocker; voltage-gated sodium channel (SCN8A) blocker; voltage-gated sodium channel (SCN9A) blocker | NMRI | 2.00 |
| 11b | NCGC00249932-01 | Emorfazone | Toothache | NMRI | 2.25 |
| 11b | NCGC00253575-01 | Arimoclomol | Niemann-Pick type C | NMRI | 2.00 |
